# Neglected tropical diseases in children: An assessment of gaps in research prioritization

**DOI:** 10.1371/journal.pntd.0007111

**Published:** 2019-01-29

**Authors:** Chris A. Rees, Peter J. Hotez, Michael C. Monuteaux, Michelle Niescierenko, Florence T. Bourgeois

**Affiliations:** 1 Division of Emergency Medicine, Boston Children’s Hospital, Harvard Medical School, Boston, Massachusetts, United States of America; 2 Texas Children's Center for Vaccine Development, National School of Tropical Medicine, Baylor College of Medicine, Houston, Texas, United States of America; 3 Computational Health Informatics Program, Boston Children's Hospital, Boston, Massachusetts, USA; S. Cuore Hospital, ITALY

## Abstract

**Background:**

Despite the known burden of neglected tropical diseases (NTDs) on child health, there is limited information on current efforts to increase pediatric therapeutic options. Our objective was to quantify and characterize research activity and treatment availability for NTDs in children in order to inform the prioritization of future research efforts.

**Methodology/Principal findings:**

We conducted a review of the World Health Organization’s (WHO) International Clinical Trials Registry Platform to assess research activity for NTDs. The burden of disease of each NTD was measured in terms of disability adjusted life years (DALYs), which was extracted from the Global Health Data Exchange. First- and second-line medications for each NTD were identified from WHO guidelines. We reviewed FDA drug labels for each medication to determine whether they were adequately labeled for use in children. Descriptive statistics, binomial tests, and Spearman’s rank order correlations were calculated to assess research activity compared to burden of disease. Children comprised 34% of the 20 million DALYs resulting from NTDs, but pediatric trials contributed just 17% (63/369) of trials studying these conditions (p<0.001 for binomial test). Conditions that were particularly under-represented in pediatric populations compared to adults included rabies, leishmaniasis, scabies, and dengue. Pediatric drug trial activity was poorly correlated with pediatric burden of disease across NTDs (Spearman’s rho = 0.41, p = 0.12). There were 47 medications recommended by the WHO for the treatment of NTDs, of which only 47% (n = 22) were adequately labeled for use in children. Of the 25 medications lacking adequate pediatric labeling, three were under study in pediatric trials.

**Conclusions/Significance:**

There is a substantial gap between the burden of disease for NTDs in children and research devoted to this population. Most medications lack adequate pediatric prescribing information, highlighting the urgency to increase pediatric research activity for NTDs with high burden of disease and limited treatment options.

## Author summary

Neglected tropical diseases are a group of poverty-associated parasitic, bacterial, and viral conditions. Collectively, they pose a substantial burden on child health, but there is limited information on current efforts to increase pediatric therapeutic options for these conditions. Understanding gaps in research activity and treatment options to reduce the global impact of neglected tropical diseases in children presents the opportunity to inform strategic initiatives and prioritize future research efforts. We analyzed trials in the World Health Organization’s International Clinical Trials Registry Platform, and found that pediatric trials comprised a disproportionately small number of drug and vaccine trials for neglected tropical diseases. Certain neglected tropical diseases, including rabies, leishmaniasis, scabies, and dengue, were particularly under-represented relative to their disease burden in children. We also determined that most medications recommended for the treatment of neglected tropical diseases lack critical data to support use in children, though few of these are currently being studied in pediatric trials. This study points to the urgent need to increase pediatric research activity for certain neglected tropical diseases that result in high disease burden and for which there are limited pediatric treatment options.

## Introduction

Neglected tropical diseases (NTDs) are a group of poverty-associated parasitic, bacterial, and viral conditions that affect more than 1.4 billion people worldwide [1]. Collectively, NTDs lead to over 500,000 deaths annually and also cause substantial morbidity, particularly among impoverished populations in low- and middle-income countries [2–4]. Several conditions disproportionately affect children compared to adults [5], and children are often simultaneously infected with multiple parasitic NTDs [6]. In sub-Saharan Africa, it is estimated that as many as 50 million children are infected with hookworm alone and in Southeast Asia, nearly 120 million pre-school and school age children require periodic treatment for soil-transmitted helminth infections [7,8].

The burden of NTDs in children is compounded by the general underrepresentation of pediatric populations in clinical trials and drug development [9]. This is principally a function of the oft limited financial incentives for drug development in children, as well as the greater scientific, ethical, and regulatory challenges in conducting trials in this population [9]. Despite the substantial and ongoing burden of NTDs on child health, there is limited information on current efforts to increase pediatric therapeutic options [5].

The objective of this study was to quantify and characterize research activity for the prevention and treatment of NTDs in children in order to inform strategic initiatives and guide the prioritization of future research efforts. We examined the alignment of research activity with burden of disease in children across NTDs and also determined the availability of pediatric prescribing information to support the use of first- and second-line therapies for NTDs in children.

## Methods

### Study design

We conducted a review of trials registered in the World Health Organization’s (WHO) International Clinical Trials Registry Platform (ICTRP) to assess research activity for NTDs. NTDs were defined based on the WHO’s 2017 list of NTDs, which includes the recent additions of chromoblastomycosis and other deep mycoses, scabies and other ectoparasites, and snakebite envenoming [10]. The Institutional Review Board at Boston Children’s Hospital determined that the study was exempt from review.

### Global burden of disease

Global disease burden for each NTD was determined based on reports of the Global Health Data Exchange [11,12]. These data are collected from 195 countries to create a comprehensive catalog of health-related data, quantifying the prevalence and impact of diseases and conditions on global public health. One of the measures provided for each disease is disability-adjusted life years (DALYs), which represent years of life lost due to disability or death. DALYs are a commonly-used metric in assessing the impact of diseases, particularly those with a chronic component [13], and have been previously used to evaluate the allocation of research and other resources across diseases [14–16]. We extracted DALYs from all regions for 2006 to 2016 and determined average yearly DALYs for each NTD. To obtain DALYs for children 0 to17 years of age, we relied on the age groups provided in the dataset and summed DALYs for children <14 years of age and three-fifths of DALYs for the age group 15 to 19 years [17].

### Clinical trials

Clinical trials for NTDs were identified in the WHO ICTRP. This publicly-accessible trial registry was created following the Ministerial Summit on Health Research in 2004 to create a single point of access for information on clinical trials globally [18]. The WHO ICTRP includes data from 17 registries from both high-income and low- and middle-income countries and provides data on roughly 430,000 trials (S1 Appendix). We also extracted data from ClinicalTrials.gov, a public trial registry administered by the U.S. National Institutes of Health [19]. Since trial records in ClinicalTrials.gov contain additional details not available in the WHO ICTRP, all trials identified in the WHO ICTRP were cross-referenced to ClinicalTrials.gov, and when available, data extracted from this registry as well.

We searched the WHO’s ICTRP Search Portal [20] on a single day (February 9, 2018) to identify all trials involving NTDs conducted between January 1, 2006 and December 31, 2016. For conditions with more than one commonly used name (e.g. Chagas and American Trypanosomiasis), all names were searched in the WHO ICTRP (S2 Appendix). Trials were included in the analysis if they studied a drug or vaccine for an NTD, were registered during the study period, were interventional, and were phase 2 to 4 trials. We excluded trials that did not primarily focus on the prevention or treatment of NTDs and those that were withdrawn or terminated. Pediatric trials were defined as those that exclusively studied children 0–17 years of age or those that also included adults but in which the mid-point of the age eligibility range was <18 years [21].

For trials selected in our final cohort, we extracted trial name, registration number, age eligibility of participants, sponsor, enrollment number, duration, randomization status, and geographic location. Sponsors were categorized as industry, government, hospital or university, or other non-profit organization. For randomization status, trials were categorized as randomized or not randomized based on the listed study design or trial description. Geographic location was determined from the listed study country or countries, and categorized based on the World Bank’s Country and Lending Group classification [22].

### Recommended therapies and pediatric labeling

Recommended treatments for NTDs were identified from WHO fact sheets published in 2017 (S3 Appendix) [23]. As there were no 2017 fact sheets for buruli ulcer, dengue, and leishmaniasis, the most recently published WHO guidelines for these conditions were used [24–26]. For chromoblastomycoses, there was no WHO-recommended first- or second-line treatments, and a widely-cited review paper was used to determine treatment options [27].

We reviewed the US Food and Drug Administration (FDA) drug labels for each WHO-recommended medication to determine status of pediatric labeling [28]. Medications were classified as adequately labeled for use in pediatric populations if labels stated that the medication was approved for pediatric use, provided dosing information for use in children, or included safety and/or efficacy information for pediatric populations [29]. If none of those were available, the medication was not considered adequately labeled for use in children.

### Trial publication

To quantify the dissemination of results from pediatric NTD trials, we searched for published articles for each of the trials in our cohort. This search was limited to trials completed by December 31, 2015 in order to allow for a minimum period of 26 months from trial completion to publication. A publication was defined as a report published in a peer-reviewed journal presenting results on at least one of the trial’s primary endpoints. We first searched for publications automatically indexed to the trial record in ClinicalTrials.gov. If none were available, we performed a systematic search in PubMed, using trial registration number, trial title, author names, institutions, and study keywords. We reviewed the abstract and, if necessary, the full text to link corresponding trials to publications based on data presented. If no publication was identified in PubMed, additional searches were performed in Embase and GoogleScholar. For industry-sponsored trials, we reviewed the company website for information on publications. For trials with more than one resulting publication, we selected the earliest publication.

### Statistical analysis

Descriptive statistics were used to characterize pediatric trials for NTDs. We used the binomial test to compare the proportion of global burden of disease that was pediatric to the proportion of clinical trials that was pediatric, collectively and for each NTD. For example, for an individual NTD, we divided the number of DALYs representing pediatric disease burden by the total number of DALYs for the condition, and compared this proportion to the proportion obtained when dividing the number of pediatric trials by the total number of trials for the condition. Conditions without burden of disease data were excluded from this analysis. As a measure of pediatric research activity for each NTD, we calculated the ratio of pediatric DALYs per pediatric trial. We used Spearman’s rho test to assess the correlation between pediatric global burden of disease in DALYs for NTDs and number of trials as well as total number of enrolled participants in all trials for each condition. To allow for representation of conditions that did not have any clinical trials, we added one trial to each condition. All analyses were conducted in Stata/SE version 13.1 (Stata Corp, College Station, TX).

## Results

**Fig 1:**
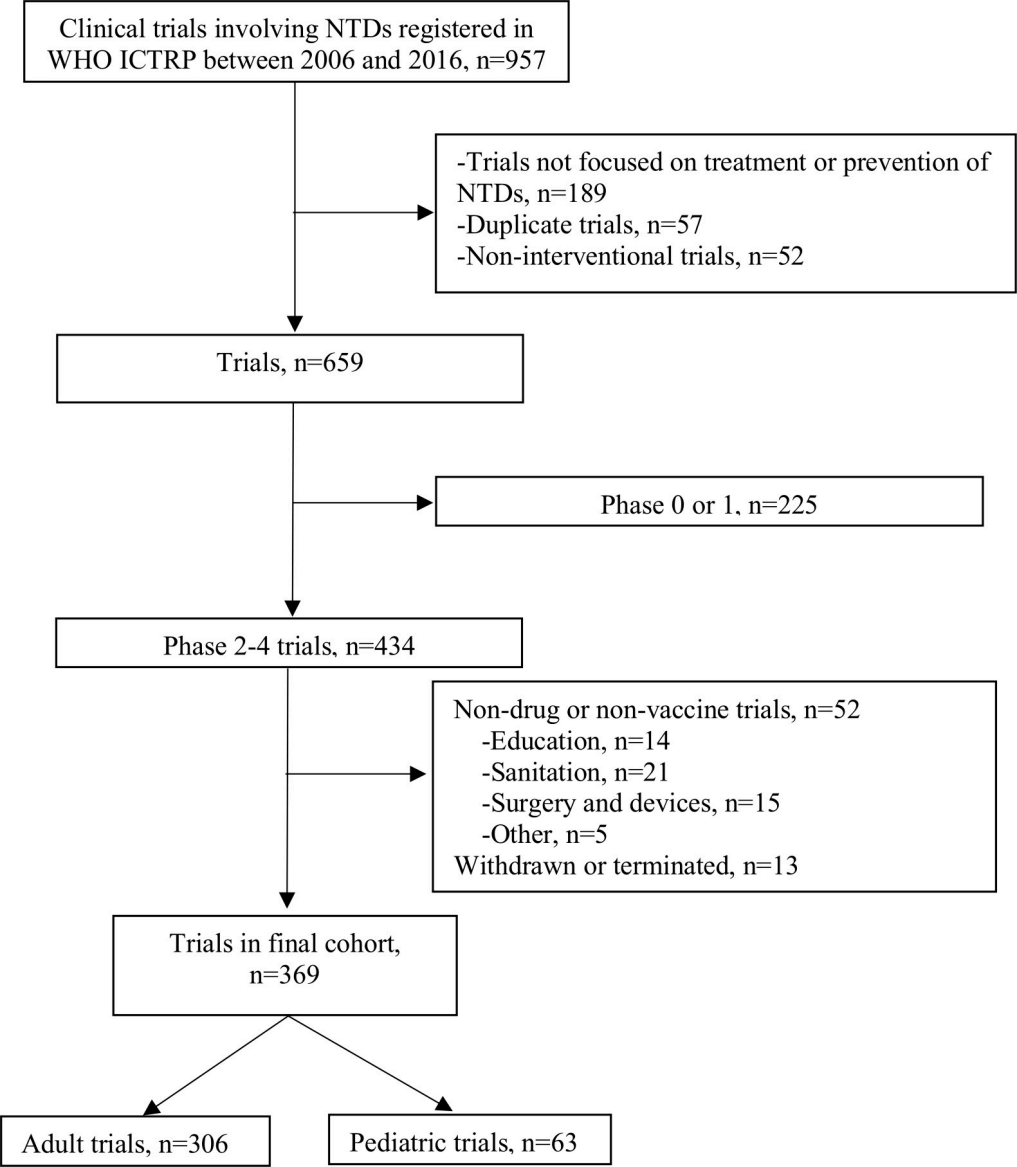
Flowchart of included pediatric trials for neglected tropical diseases. LEGEND: *NTDs: neglected tropical diseases, WHO ICTRP: World Health Organization International Clinical Trials Registry Platform.

There were 369 trials studying the treatment or prevention of NTDs registered in the WHO ICTRP from 2006 to 2016 that met inclusion criteria (Fig 1) (S1 Table). Of these, 63 (17%) were pediatric trials and 306 (83%) adult trials. Of the 63 pediatric trials, 54 (86%) studied exclusively children 0 to 18 years of age. The remaining 9 trials had age eligibilities ranging from 0 to 22 years.

### Pediatric trial characteristics

Among the 63 pediatric trials, two-thirds (n = 42) were drug trials and a third (n = 21) were vaccine trials, of which the majority (n = 16) were dengue vaccine trials. 85% (n = 52) of the trials were randomized and 40% (n = 25) were funded by industry sponsors (Table 1). The median enrollment was 315 participants and the mean trial duration 2.3 years. Of the 60 trials that provided information on study location, 65% (n = 36) were conducted in a single country, 25% (n = 15) in two countries, and 15% (n = 9) in three or more countries. Among these trials, 32% (n = 19) included sites in sub-Saharan Africa, 30% (n = 18) in Latin America and the Caribbean, 22% (n = 13) in East Asia and the Pacific, 7% (n = 4) in South Asia, 5% (n = 3) in Europe and Central Asia, 3% (n = 2) in the Middle East and North Africa, and 2% (n = 1) in North America.

**Table 1:** Characteristics of clinical trials for neglected tropical diseases.

Condition	Adult Trials, n	Pediatric Trials, n	Characteristics of Pediatric Trials					
			Drug Trials, n (%)	Vaccine Trials, n (%)	Randomized Trials, n (%)	Industry Funded, n (%)	Enrollment, median	Trial Duration in Years, mean*
Dengue and chikungunya	42	21	5 (23.8)	16 (76.2)	20 (95.2)	17 (80.9)	504	3.3
Schistosomiasis	6	14	12 (85.7.0)	2 (14.3)	12 (85.7)	2 (14.3)	490	1.6
Soil Transmitted Helminthiases	12	9	9 (100.0)	0	7 (77.8)	2 (22.2)	350	1.3
Leishmaniasis	87	5	5 (100.0)	0	3 (60.0)	0	80	2.9
Rabies	33	4	0	4 (100.0)	2 (50.0)	3 (75.0)	246	0.8
Chagas	19	2	2 (100.0)	0	1 (50.0)	1 (50.0)	205	2.0
Scabies and Other Ectoparasites	18	2	1 (50.0)	1 (50.0)	1 (50.0)	0	266	1.4
Trachoma	11	2	2 (100.0)	0	2 (100.0)	0	1,657	6.1
Yaws	3	2	2 (100.0)	0	2 (100.0)	0	419	1.3
Human African Trypanosomiasis	5	1	1 (100.0)	0	0	0	125	3.2
Taeniasis/Cysticercosis	5	1	1 (100.0)	0	1 (100.0)	0	97	1.6
Dracunculiasis	0	0	-	-	-	-	-	-
Echinococcosis	1	0	-	-	-	-	-	-
Foodborne Trematodiases	1	0	-	-	-	-	-	-
Leprosy	18	0	-	-	-	-	-	-
Lymphatic filariasis	16	0	-	-	-	-	-	-
Mycetoma, chromoblastomycosis, and other deep mycoses	6	0	-	-	-	-	-	-
Onchocerciasis	8	0	-	-	-	-	-	-
Buruli Ulcer	3	0	-	-	-	-	-	-
Snake Bite Envenomation	12	0	-	-	-	-	-	-
**Total**	**306**	**63**	**42 (66.6)**	**21 (33.3)**	**52 (85.3)**	**25 (39.7)**	**315**	**2.3**

*Completion dates for the calculation of trial duration were missing for 4 trials on dengue, 2 trials on schistosomiasis, and 1 trial each on leishmaniasis, rabies, trachoma, and soil transmitted helminthiases.

There were 45 trials that were completed as of December 31, 2015, allowing for a minimum follow up period of 26 months and mean follow up of 44 months. Published articles reporting on the trial’s primary outcome were available for 80% (n = 36) of these trials, with an average of 1.4 publications per trial (range 1–9). Nearly two-thirds (65%, n = 33) of the published articles were published in open access format.

### Pediatric research activity compared to global burden of disease

**Fig 2:**
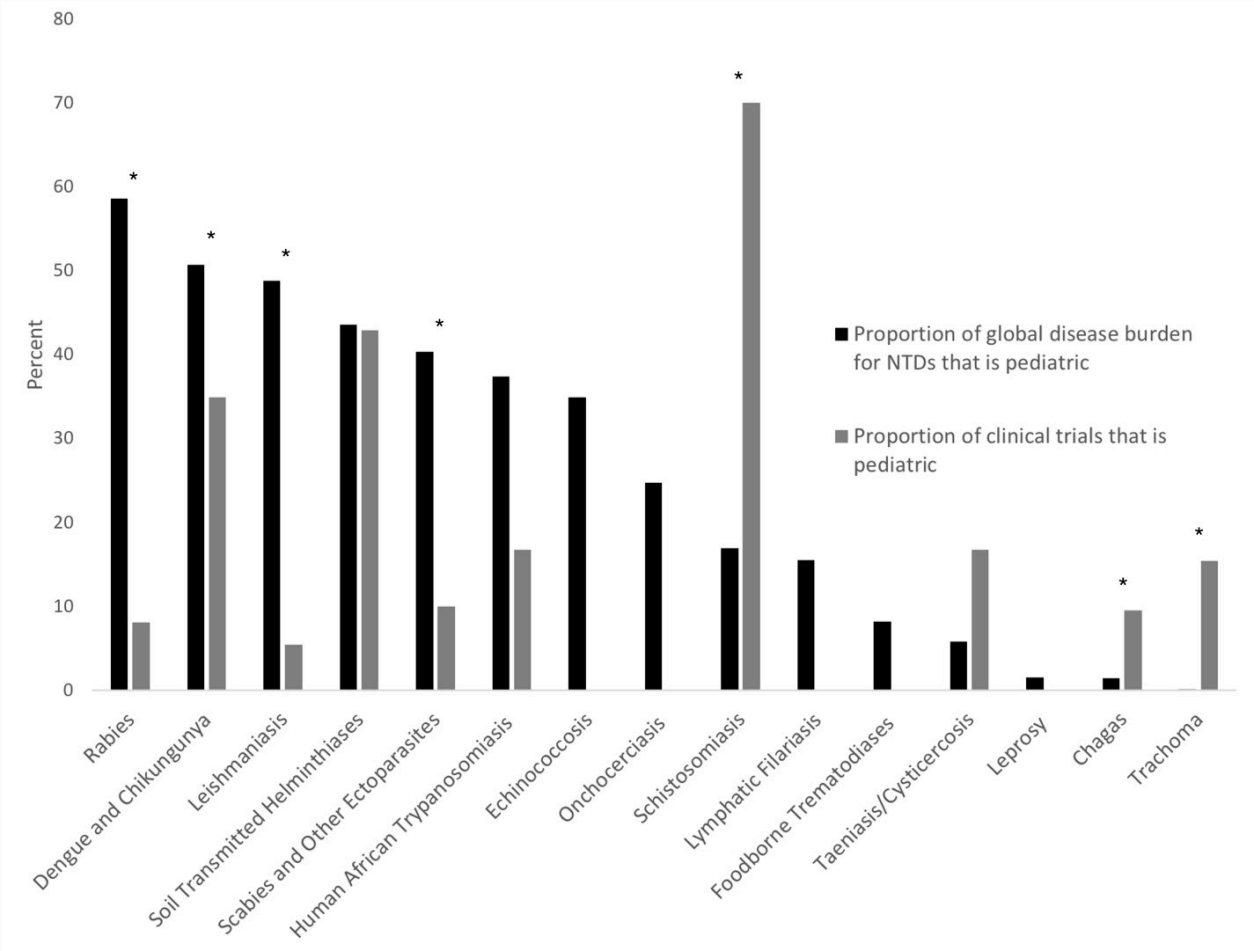
Comparison of proportion of clinical trials that is pediatric to proportion of global disease burden that is pediatric for neglected tropical diseases. LEGEND: *Indicates p-values <0.05 (rabies <0.001, dengue and chikungunya 0.016, leishmaniasis <0.001, scabies and other ectoparasites 0.005, schistosomiasis <0.001, chagas 0.035, trachoma, <0.001) *†*Buruli ulcer, mycetoma, snakebite envenoming, and yaws are not included as the Global Health Data Exchange does not include DALYs for these diseases. *‡*Dracunculiasis was not included as there are no adult registered trials for this condition.

There were 15 NTDs with available DALYs. These NTDs resulted in approximately 20.3 million DALYs averaged over the study period, of which 6.8 million (34%) were attributable to disease in children. However, pediatric trials for NTDs comprised just 17% (63/369) of phase 2–4 trials for NTDs (p<0.001 for binomial test comparing proportions of pediatric DALYs and trials) (Fig 2). Several conditions were particularly under-represented when considering burden of disease and research allocation between children and adults, including rabies, leishmanisias, scabies, and dengue. Others appeared to have higher proportions of research activity in children, including schistosomiasis, taeniasis, trachoma, and Chagas disease. In the case of schistosomiasis, which had the highest proportion of pediatric trials, the large number of pediatric trials represented primarily testing of a single product, praziquantel (10 of the 14 pediatric trials).

Table 2 presents the ratios of pediatric DALYs per pediatric trial for each of the NTDs as a measure of research activity committed to each of the conditions. The ratios of DALYs per trial were least favorable for scabies, onchocerciasis, and lymphatic filariasis. When evaluating ratios of DALYs per participant, leishmaniasis, rabies, and scabies were comparatively neglected. The overall ratio of DALYs per trial across all NTDs was worst for children compared to adults (89 DALYs per pediatric trial vs. 41 DALYs per adult trial).

**Table 2:** Comparison of disability adjusted life years and clinical trials for each neglected tropical disease among children.

Condition*	Pediatric Global Burden of Disease (per 1,000 DALYs)	Pediatric Trials, n	DALYs per Trial (1,000 DALYs)^a^	DALYs per Participant^b^
Dengue and Chikungunya	1,208	22	53	15
Schistosomiasis	371	14	25	52
Soil Transmitted Helminthiases	1,578	9	158	187
Leishmaniasis	721	5	120	1,689
Rabies	601	3	150	1,114
Chagas	3	2	1	8
Scabies and other Ectoparasites	1,507	2	502	2,833
Trachoma	0	2	0	0.1
Human African Trypanosomiasis	118	1	59	947
Taeniasis/Cysticercosis	31	1	16	316
Dracunculiasis	0	0	0	-
Echinococcosis	60	0	60	-
Foodborne Trematodiases	139	0	139	-
Leprosy	0	0	0	-
Lymphatic Filariasis	242	0	242	-
Onchocerciasis	273	0	273	-
**Total**	**6,855**	**61**	**89**	**196**

*Buruli ulcer, mycetoma, snakebite envenoming, and yaws are not included as the Global Health Data Exchange does not include DALYs for these diseases.

^**a**^Calculated by dividing the pediatric global burden of disease (per 1,000 DALYs) by the number of pediatric trials for each NTD. To allow for representation of conditions that do not have any clinical trials, we added 1 trial to each condition.

^**b**^Calculated by dividing the pediatric global burden of disease (per 1,000 DALYs) by the number of trial participants for each NTD.

Across NTDs, pediatric trial activity was moderately correlated with pediatric global burden of disease (Spearman’s rho = 0.60, p = 0.013). When assessing correlation based on trial enrollment, the correlation was slightly less (Spearman’s rho = 0.49, p = 0.15). We also examined the correlation limited to drug trials, which revealed a poor correlation between trial activity and disease burden (Spearman’s rho = 0.41, p = 0.12). However, pediatric vaccine trial activity was moderately correlated with pediatric global burden of disease (Spearman’s rho = 0.53, p = 0.03).

### Available medications for the treatment of NTDs

There were 47 medications recommended by the WHO for first- and second-line treatment of NTDs, of which 22 (47%), representing 15 conditions, were adequately labeled for use in children (S2 Table). These 22 drugs included 8 for use in infants and children less than 2 years of age, 8 for children 2 to 10 years of age, and 6 for children age 10 and older. Only 7 of the 47 (15%) medications were available in pediatric formulations. Of the 25 recommended medications for which there was not adequate pediatric labeling, only three had pediatric trials in our cohort (nifurtimox, meglumine, and pentavalent antimonials). In addition, two of the NTDs—fascioliasis (one of the foodborne trematodiases) and mycetoma, chromoblastomycosis, and other deep mycoses—had no medications that were adequately labeled for use in children. There were no pediatric trials in our cohort studying these conditions.

Table 3 presents the drugs and vaccines studied in pediatric NTD trials. Of the 11 NTDs for which there were interventional trials, 7 (64%) included trials for drugs or vaccines not currently labeled for use in children.

**Table 3:** Drugs and vaccines studied in pediatric trials for neglected tropical diseases.

Condition	WHO-Recommended Therapies	Drugs Studied	Vaccines Studied
Chagas	• Benznidazole* • Nifurtimox	• Benznidazole • Nifurtimox	
Dengue and Chikungunya	• Supportive care*	• Hypertonic sodium lactate and Ringer's lactate • Anti-CHIKV hyperimmune immunoglobulins • Zinc • Early corticosteroid therapy Vitamin E	• Tetravalent Dengue Vaccine Chimeric tetravalent dengue-serotype 1, 2, 3, 4 • Live, attenuated dengue serotype 1, 2, 3, and 4 • Takeda's Tetravalent Dengue Vaccine Candidate • CYD Dengue Vaccine • CYD Dengue Vaccine-5 dose formulation • Tetravalent Dengue Vaccine
Human African Trypanosomiasis	• Pentamidine* • Suramin • Eflornithine* and Nifurtimox • Melarsoprol	• Fexinidazole	
Leishmaniasis	• Meglumine antimoniate • Sodium stibogluconate • Amphotericin B deoxycholate* • Pentamidine isethionate* • Paromomycin ointment • Fluconazole* • Topical methylbenzethonium • Topical ketoconazole* • Miltefosine* • Pentavalent antimonials • Pentoxifylline • Liposomal amphotericin B*	• Miltefosine • Meglumine antimoniate • Azithromycin • Amphotericin B-deoxycholate	
Rabies	• Immediate vaccination • Rabies immune globulin	• Human rabies immunoglobulin	• Purified Vero Rabies Vaccine Serum Free and Imovax Human Diploid Cell Vaccine • Rabipur • CL184
Scabies and Other Ectoparasites	• Permethrin cream* • Malathion cream* • Benzyl benzoate emulsion cream • Sulphur ointment • Ivermectin*	• KD-357 I• vermectin • Permethrin	
Schistosomiasis	• Praziquantel* • Oxamniquine	• Praziquantel • Biltricide (racemate praziquantel) oral tablets • Racemate Praziquantel ODT • Levo Praziquantel ODT • Artemether-lumefantrine combination plus albendazole • Artemether-lumefantrine plus Praziquantel plus Albendazole • Albendazole plus Praziquantel • Moxidectin • Synriam • Artesunate+Sulfamethoxy-pyrazine/pyrimethamine • Albendazole + praziquantel • Mebendazole + praziquantel • Sulfadoxine-pyrimethamine • Piperaquine • Albendazole • Praziquantel • Praziquantel and Arachidonic acid	• Bilhvax vaccine-Sh28GST • MVA85A
Soil Transmitted Helminthiases	• Pyrantel • Mebendazole* • Levamisole • Piperazine • Albendazole* • Flubendazole • Ivermectin*	• Albendazole • Mebendazole • Albendazole and Vitamin C • Oxantel pamoate • Pyrantel-oxantel + mebendazole • Ivermectin • Albendazole + Ivermectin • Mebendazole + Ivermectin	
Taeniasis/Cysticercosis	• Praziquantel* • Niclosamide	• Albendazole	
Trachoma	• Azithromycin*	• Azithromycin	
Yaws	• Azithromycin* • Benzathine penicillin*	• Azithromycin • Penicillin-G-benzathine + Azithromycin	

*Adequately labeled for use in children.

## Discussion

Children comprise a significant proportion of the global burden of disease resulting from NTDs, but research activity dedicated to preventing and treating these diseases in children lags behind. Certain NTDs are particularly under-represented in pediatric research when compared to adults, presenting areas of opportunity for focused investment to improve the health of children in low- and middle-income countries. Furthermore, trials for NTDs conducted in children are not well aligned with the distribution of disease burden in this population. Overall, less than half of WHO-recommended therapies for NTDs are adequately labeled for use in children, highlighting the urgent need to increase pediatric research activity for NTDs, with prioritization of diseases that represent significant burden and lack adequate treatment options.

Our findings define several NTDs that have been understudied in pediatric populations based on the proportion of global burden comprised by children: rabies, leishmaniasis, scabies, and dengue. Rabies, a lethal viral infection acquired through animal bites and leading to rapidly progressive encephalitis [30], is a rare condition with only 289 prevalent cases in the Global Burden of Disease database. Leishmaniasis, by comparison, is a common parasitic infection that is acquired from several species of sandflies and leads to both self-healing cutaneous lesions and multiorgan, visceral disease, with the latter leading to over 62,000 deaths annually [31]. In contrast to studies in adults that showed leishmaniasis was one of the most-studied NTDs [32] our study showed that it is relatively under-represented in pediatric populations. Recent evidence suggests that the response to pentavalent antimonial, the medication historically used to treat visceral leishmaniasis, is declining [33]. As such, oral miltefesone and amphotericin B are increasingly being used as treatment for leishmanisis. However, large trials assessing the efficacy of these drugs in children are lacking [33], and there were no trials in our cohort assessing these two medications. Scabies is caused by mites leading to a pruritic rash and can lead to serious sequelae due to secondary impetigo and other bacterial infections, including glomerulonephritis and rheumatic heart disease [34–36]. We found that research for this condition is under-represented in children, though this may be of lesser concern given the availability of several treatment options for use in children. Dengue also has a high prevalence, providing opportunity for pediatric-specific study, with over 100 million symptomatic cases annually and 2.4 million prevalent pediatric cases in 2016 alone [12,37]. Dengue is an arthropod-borne viral disease that leads to mild febrile illnesses to dengue shock syndrome in severe cases [25]. Though the treatment for dengue is supportive in nature, dengue vaccines are under development [38,39]. Moving forward, ongoing assessments of pediatric research activity for NTDs are required to monitor areas of greatest need as new drugs and vaccines become available.

Our findings likely do not fully capture the deficiency in pediatric clinical trials, as reports indicate that the DALYs assigned to many of the NTDs disproportionately affecting children may represent severe underestimates [40]. For example, the disease burden of schistosomiasis does not fully consider its underlying chronic morbidities [41], while estimates for hookworm infection do not fully consider the impact of moderate and severe iron deficiency anemia [42]. Moreover, many of the disfiguring NTDs, such as cutaneous leishmaniasis, do not adequately incorporate profound and lifelong mental health challenges due to social stigma [43].

Similar to our findings, previous studies have shown that many pediatric conditions are understudied and that pediatric research is only moderately correlated with pediatric disease burden [17,44]. These studies indicate that the alignment of research activity with pediatric disease burden is lowest in low- and middle-income countries, which is where the largest burden of NTDs lies [1,3]. Another study examining pediatric and adult research for 13 NTDs showed that there was generally poor correlation between global disease burden and the number of clinical trials or trial participants [32]. Our findings add to this work, demonstrating that pediatric trial activity for NTDs is not optimally aligned with pediatric treatment needs or burden of disease.

By definition, NTDs have long been recognized as receiving less research investment than other conditions in low- and middle-income countries [15]. However, a number of initiatives have been implemented over the past decade to control and eliminate NTDs. The WHO, in a report on efforts to control and eliminate NTDs, has advocated for preventive chemotherapy through mass drug administration in areas where NTDs are endemic, intensifying case management, and the provision of clean water and sanitation [45]. Furthermore, with the support of donors, non-governmental organizations, and pharmaceutical companies, the London Declaration on Neglected Tropical Diseases was signed in 2012, which presents a commitment to “control, eliminate, or eradicate” 10 NTDs by 2020 [46]. Children are included in these efforts with specific strategies outlined for a number of NTDs in pediatric populations. Our study provides data to further guide investments and resource allocation in order to target diseases that contribute the greatest disease burden and that lack adequate pediatric treatment options.

Our study has some limitations. While we attempted to create a comprehensive list of all trials conducted for NTDs, it is possible that some trials were not registered and were not included in the WHO ICTRP. In addition, as phase 1 trials are not consistently reported in the trial registries and are less likely to translate to clinical care, they were excluded from the analysis in this study. We used DALYs to measure disease burden, but DALYs may be dependent on country-specific reporting and estimates. They are also not a perfect measure of clinical need, since there may be less urgency to develop new pediatric therapies for certain NTDs, such as scabies, if therapies already exist. However, DALYs are a robust metric for measuring morbidity and mortality across country income groups and are commonly used in these types of analyses [14–16]. Lastly, NTDs predominately affect patients in low- and middle-income countries and we examined prescribing information based on medication labels provided by the FDA as it is the single unified, publicly-available source of these data that we could identify. However, this is likely an accurate representation of the pediatric prescribing data available to clinicians practicing in middle- and low-income countries.

### Conclusions

There is a substantial gap between the burden of disease for NTDs in children and research devoted to this population, with certain conditions particularly under-represented compared to adult research investments. Most first- and second-line medications recommended for the treatment of these conditions lack adequate pediatric prescribing information, highlighting the urgency to increase pediatric research activity for NTDs with high disease burden and limited treatment options.

## Supporting information

S1 Checklist (DOCX)

S1 Appendix List of trial registries included in the WHO ICTRP. (DOCX)

S2 Appendix Search terms used to extract trials from the WHO ICTRP and ClinicalTrials.gov. (DOCX)

S3 Appendix Source for first- and second-line medications for the treatment of each of the WHO-designated neglected tropical diseases as accessed on November 28, 2017. (DOCX)

S1 Table Phase 2–4 interventional trials of drugs and vaccines for neglected tropical diseases registered in the WHO ICTRP, 2006 to 2016. (DOCX)

S2 Table World Health Organization (WHO) recommended medications for the treatment of neglected tropical diseases in children *Second line therapy. (DOCX)

## References

[1] Hotez PJ, Molyneux DH, Fenwick A, Kumaresan J, Sachs SE, Sachs JD, et al Control of neglected tropical diseases. N Engl J Med. 2007;357:1018–27. 10.1056/NEJMra064142 17804846

[2] Hotez PJ, Molyneux DH, Fenwick A, Ottesen E, Sachs SE, Sachs JD. Incorporating a rapid-impact package for neglected tropical diseases with programs for HIV/AIDS, tuberculosis, and malaria: A comprehensive pro-poor health policy and strategy for the developing world. PLoS Med. 2006;3:576–84. 10.1371/journal.pmed.0030102 PMC1351920 16435908

[3] Molyneux DH, Hotez PJ, Fenwick A. “Rapid-impact interventions”: How a policy of integrated control for Africa’s neglected tropical diseases could benefit the poor. PLoS Med. 2005;2:1064–70. 10.1371/journal.pmed.0020336 PMC1253619 16212468

[4] Naghavi M, Abajobir AA, Abbafati C, Abbas KM, Abd-Allah F, Abera SF, et al Global, regional, and national age-sex specifc mortality for 264 causes of death, 1980–2016: A systematic analysis for the Global Burden of Disease Study 2016. Lancet. 2017;390:1151–210. 10.1016/S0140-6736(17)32152-9 28919116 PMC5605883

[5] Barry MA, Murray KO, Hotez PJ, Jones KM. Impact of vectorborne parasitic neglected tropical diseases on child health. Arch Dis Child. 2016;101:640–7. 10.1136/archdischild-2015-308266 26921274

[6] Steinmann P, Utzinger J, Du ZW ZX. Multiparasitism a neglected reality on global, regional and local scale. Adv Parasitol. 2010;73:21–50. 10.1016/S0065-308X(10)73002-5 20627138

[7] Hotez PJ, Kamath A. Neglected tropical diseases in sub-Saharan Africa: Review of their prevalence, distribution, and disease burden. PLoS Negl Trop Dis. 2009;3:2–11. 10.1371/journal.pntd.0000412 PMC2727001 19707588

[8] Hotez PJ, Bottazzi ME, Strych U, Chang LY, Lim YAL, Goodenow MM, et al Neglected Tropical Diseases among the Association of Southeast Asian Nations (ASEAN): Overview and Update. PLoS Negl Trop Dis. 2015;9:1–15. 10.1371/journal.pntd.0003575 PMC4400050 25880767

[9] Hudgins JD, Bacho MA, Olsen KL, Bourgeois FT. Pediatric drug information available at the time of new drug approvals: A cross-sectional analysis. Pharmacoepidemiol Drug Saf. 2018;27:161–7. 10.1002/pds.4351 29148107

[10] World Health Organization. Neglected Tropical Diseases. [Internet]. 2017 [cited 2017 Nov 14]. Available from: http://www.who.int/neglected_diseases/diseases/en/

[11] Abajobir AA, Abate KH, Abbafati C, Abbas KM, Abd-Allah F, Abdulkader RS, et al Global, regional, and national disability-adjusted life-years (DALYs) for 333 diseases and injuries and healthy life expectancy (HALE) for 195 countries and territories, 1990–2016: a systematic analysis for the Global Burden of Disease Study 2016. Lancet. 2017;390:1260–344. 10.1016/S0140-6736(17)32130-X 28919118 PMC5605707

[12] Global Health Data Exchange. Search Tool. [Internet]. [cited 2017 Nov 14]. Available from: http://ghdx.healthdata.org/gbd-results-tool

[13] World Health Organization. Metrics: Disability-Adjusted Life Years. [Internet]. [cited 2018 Jan 30]. Available from: http://www.who.int/healthinfo/global_burden_disease/metrics_daly/en/

[14] Yoong SL, Hall A, Williams CM, Skelton E, Oldmeadow C, Wiggers J, et al Alignment of systematic reviews published in the Cochrane database of systematic reviews and the database of abstracts and reviews of effectiveness with global burden-of-disease data: A bibliographic analysis. J Epidemiol Community Health. 2015;69:708–14. 10.1136/jech-2014-205389 25888595 PMC4483792

[15] Isaakidis P, Swingler GH, Pienaar E, Volmink J, Ioannidis JPA. Relation between burden of disease and randomised evidence in sub-Saharan Africa: Survey of research. Br Med J. 2002;324:702–5. 11909786 10.1136/bmj.324.7339.702 PMC99053

[16] Bhaumik S, Karimkhani C, Czaja C, Williams H, Rani M, Nasser M, et al Identifying gaps in research prioritization: The global burden of neglected tropical diseases as reflected in the Cochrane database of systematic reviews. J Fam Med Prim Care. 2015;4:507. 10.4103/2249-4863.174266 PMC4776600 26985407

[17] Bourgeois FT, Olson KL, Ioannidis JPA, Mandl KD. Association Between Pediatric Clinical Trials and Global Burden of Disease. Pediatrics. 2014;133:78–87. 10.1542/peds.2013-2567 24344112 PMC3876184

[18] World Health Organization. About the WHO ICTRP. [Internet]. [cited 2018 Jan 16]. Available from: http://www.who.int/ictrp/about/en/

[19] United States National Library of Medicine. ClinicalTrials.gov [Internet]. [cited 2017 Nov 16]. Available from: https://clinicaltrials.gov/ct2/search/advanced?cond=&term=&state1=&cntry1=

[20] World Health Organization. WHO ICTRP Search Portal. [Internet]. [cited 2018 Jan 16]. Available from: http://apps.who.int/trialsearch/

[21] Bourgeois FT, Murthy S, Pinto C, Olson KL, Ioannidis JPA, Mandl KD. Pediatric Versus Adult Drug Trials for Conditions With High Pediatric Disease Burden. Pediatrics. 2012;130:285–92. 10.1542/peds.2012-0139 22826574 PMC3408692

[22] Worldbank. Country and lending groups [Internet]. Worldbank. 2013 [cited 2018 Jan 10]. p. 1. Available from: https://datahelpdesk.worldbank.org/knowledgebase/articles/906519

[23] World Health Organization. Neglected Tropical Diseases: Fact Sheets Related to NTD [Internet]. 2017 [cited 2017 Nov 30]. Available from: http://www.who.int/neglected_diseases/mediacentre/factsheet/en/

[24] World Health Organization. Treatment of Mycobacterium Ulcerans Disease (Buruli Ulcer) [Internet]. 2012 [cited 2017 Nov 28]. Available from: http://apps.who.int/iris/bitstream/10665/77771/1/9789241503402_eng.pdf?ua=1

[25] World Health Organization. Dengue: Guidelines for Diagnosis, Treatment, Prevention and Control [Internet]. 2009 [cited 2017 Nov 28]. Available from: http://www.who.int/tdr/publications/documents/dengue-diagnosis.pdf 23762963

[26] World Health Organization. Control of the leishmaniases [Internet]. 2010 [cited 2017 Nov 28]. Available from: http://apps.who.int/iris/bitstream/10665/44412/1/WHO_TRS_949_eng.pdf

[27] Queiroz-Telles F, Esterre P, Perez-Blanco M, Vitale RG, Salgado CG, Bonifaz A. Chromoblastomycosis: an overview of clinical manifestations, diagnosis and treatment. Med Mycol. 2009;47:3–15. 10.1080/13693780802538001 19085206

[28] United States Food and Drug Administration. Drugs@FDA: FDA Approved Drug Products [Internet]. [cited 2017 Nov 16]. Available from: https://www.accessdata.fda.gov/scripts/cder/daf/index.cfm

[29] Sachs AN, Avant D, Lee CS, Rodriguez W, Murphy MD. Pediatric information in drug product labeling. JAMA. 2012;307:1914–5. 10.1001/jama.2012.3435 22570457

[30] Willoughby RE. Rabies: Rare Human Infection—Common Questions. Infect Dis Clin North Am. 2015;29:637–50. 10.1016/j.idc.2015.07.006 26384549

[31] Al-Salem W, Herricks JR, Hotez PJ. A review of visceral leishmaniasis during the conflict in South Sudan and the consequences for East African countries. Parasites and Vectors. 2016;9:1–11. 10.1186/s13071-015-1291-6 27549162 PMC4994383

[32] Kappagoda S, Ioannidis JPA. Neglected tropical diseases: Survey and geometry of randomised evidence. BMJ. 2012;345:1–15. 10.1136/bmj.e6512 PMC3478233 23089149

[33] Sundar S, Agarwal D. Visceral Leishmaniasis—Optimum Treatment Options in Children. Pediatr Infect Dis J. 2018;37:492–4. 10.1097/INF.0000000000001885 29280784 PMC5990428

[34] Diamantis SA, Morrell DS, Burkhart CN. Pediatric infestations. Pediatr Ann. 2009;38:326–32. 19588676 10.3928/00904481-20090521-05

[35] Thornley S, Marshall R, Jarrett P, Sundborn G, Reynolds E, Schofield G. Scabies is strongly associated with acute rheumatic fever in a cohort study of Auckland children. J Paediatr Child Health. 2018;54:625–32. 10.1111/jpc.13851 29442387

[36] Yeoh DK, Anderson A, Cleland G, Bowen AC. Are scabies and impetigo “normalised”? A cross-sectional comparative study of hospitalised children in northern Australia assessing clinical recognition and treatment of skin infections. PLoS Negl Trop Dis. 2017;11:1–16. 10.1371/journal.pntd.0005726 PMC5510902 28671945

[37] Bhatt S, Gething PW, Brady OJ, Messina JP, Farlow AW, Moyes CL, et al The global distribution and burden of dengue. Nature. 2013;496:504–7. 10.1038/nature12060 23563266 PMC3651993

[38] Capeding MR, Tran NH, Hadinegoro SRS, Ismail HIHM, Chotpitayasunondh T, Chua MN, et al Clinical efficacy and safety of a novel tetravalent dengue vaccine in healthy children in Asia: A phase 3, randomised, observer-masked, placebo-controlled trial. Lancet. 2014;384:1358–65. 10.1016/S0140-6736(14)61060-6 25018116

[39] Villar L, Dayan GH, Arredondo-García JL, Rivera DM, Cunha R, Deseda C, et al Efficacy of a tetravalent dengue vaccine in children in Latin America. N Engl J Med. 2015;372:113–23. 10.1056/NEJMoa1411037 25365753

[40] Herricks JR, Hotez PJ, Wanga V, Coffeng LE, Haagsma JA, Basáñez MG, et al The global burden of disease study 2013: What does it mean for the NTDs? PLoS Negl Trop Dis. 2017;11:1–21. 10.1371/journal.pntd.0005424 PMC5542388 28771480

[41] King CH GA. Underestimation of the global burden of schistosomiasis. Lancet. 2018;391:307–8. 10.1016/S0140-6736(18)30098-9 29413041

[42] Bartsch SM, Hotez PJ, Asti L, Zapf KM, Bottazzi ME, Diemert DJ, et al The Global Economic and Health Burden of Human Hookworm Infection. PLoS Negl Trop Dis. 2016;10: e0004922 10.1371/journal.pntd.0004922 27607360 PMC5015833

[43] Bailey F, Mondragon-Shem K, Hotez P, Ruiz-Postigo JA, Al-Salem W, Acosta-Serrano Á, et al A new perspective on cutaneous leishmaniasis—Implications for global prevalence and burden of disease estimates. PLoS Negl Trop Dis. 2017;11:2–6. 10.1371/journal.pntd.0005739 PMC5552022 28796782

[44] Joseph PD, Caldwell PHY, Barnes EH, Craig JC. Disease burden-research match? Registered trials in child health from low- and middle-income and high-income countries. J Paediatr Child Health. 2017;53:667–74. 10.1111/jpc.13537 28383200

[45] World Health Organization. Working to overcome the global impact of neglected tropical diseases [Internet]. First WHO report on neglected tropical diseases. 2010 [cited 2018 Jun 26]. Available from: http://apps.who.int/iris/bitstream/handle/10665/44440/9789241564090_eng.pdf?sequence=1

[46] London Declaration on Neglected Tropical Diseases. [Internet]. 2012 [cited 2018 Jun 26]. Available from: http://unitingtocombatntds.org/london-declaration-neglected-tropical-diseases/

