# Supplementary material for: Neglected tropical diseases in children: An assessment of gaps in research prioritization
Source: PLoS Negl Trop Dis. 2019 Jan 29;13(1):e0007111. doi: 10.1371/journal.pntd.0007111 (PMC6368333; doi:10.1371/journal.pntd.0007111)
Supplement: S1 Appendix — (DOCX) [file pntd.0007111.s002.docx]

1. Australian New Zealand Clinical Trials Registry (ANZCTR)
2. Brazilian Clinical Trials Registry (ReBec)
3. Chinese Clinical Trial Register (ChiCTR)
4. Clinical Research Information Service (CRiS), Republic of Korea
5. ClinicalTrials.gov
6. Clinical Trials Registry - India (CTRI)
7. Cuban Public Registry of Clinical Trials (RPCEC)
8. EU Clinical Trials Register (EU-CTR)
9. German Clinical Trials Register (DRKS)
10. Iranian Registry of Clinical Trials (IRCT)
11. ISRCTN.org
12. Japan Primary Registries Network (JPRN)
13. Pan African Clinical Trial Registry (PACTR)
14. Peruvian Clinical Trials Registry (REPEC)
15. Sri Lanka Clinical Trials Registry (SLCTR)
16. Thai Clinical Trials Register (TCTR)
17. The Netherlands National Trial Register (NTR)
