# Supplementary material for: Neglected tropical diseases in children: An assessment of gaps in research prioritization
Source: PLoS Negl Trop Dis. 2019 Jan 29;13(1):e0007111. doi: 10.1371/journal.pntd.0007111 (PMC6368333; doi:10.1371/journal.pntd.0007111)
Supplement: S2 Appendix — (DOCX) [file pntd.0007111.s003.docx]

| **Condition** | **Search Terms** |
| --- | --- |
| Buruli ulcer | “Buruli ulcer” “Buruli” |
| Chagas | “Chagas” “American trypanosomiasis” “Trypanosomiasis” |
| Dengue and chikungunya | “Dengue”  “Breakbone fever” “Dengue hemorrhagic fever” “Dengue shock syndrome” |
| Dracunculiasis | “Dracunculiasis” “guinea worm” “guinea worm disease” “Dracunculus” |
| Echinococcosis | “Echinococcosis” “Echinococcus” “Hydatid” |
| Foodborne Trematodiases | “Foodborne trematodiases” “Trematode” “Trematodiases” “Fascioliasis” “Opisthorchiasis” “Clonorchiasis” “Paragonimiasis” |
| Human African Trypanosomiasis | “Human African Trypanosomiasis” “Sleeping sickness” “Gambiense trypanosomiasis” “Rhodesiense trypanosomiasis” |
| Leishmaniasis | “Leishmaniasis” “Kala azar” ”Visceral Leishmaniasis” “Cutaneous Leishmaniasis” |
| Leprosy | “Leprosy” “Hansen's disease” |
| Lymphatic filariasis | “Lymphatic filariasis” “Filariasis” “*Wuchereria bancrofti*” |
| Mycetoma, chromoblastomycosis, and other deep mycoses | “Mycetoma” “Chromoblastomycosis” “Deep mycoses” “Mycoses” |
| Onchocerciasis | “Onchocerciasis” “River blindness” |
| Rabies | “Rabies” |
| Scabies and other ectoparasites | “Scabies” “Ectoparasite” |
| Schistosomiasis | “Schistosomiasis” “Bilharzia” “Snail fever” |
| Soil Transmitted Helminthiases | “Soil transmitted helminthiases” “helminths” “Intestinal nematode infection” |
| Ascariasis | “Ascariaisis” “Roundworm” |
| Hookworm | “Hookworm” “Uncinariasis” “*Ancylostoma duodenale*” “*Necator americanus*” |
| Trichuriasis | “Trichuriasis” “whipworm” |
| Taeniasis/Cysticercosis | “Taeniasis” “Cysticercosis” “Taenia” “Cysticerciasis” “Neurocysticercosis” |
| Trachoma | “Trachoma” “Chlamydial conjunctivitis” |
| Yaws | “Yaws” “*Treponema pertenue*” |
