# Supplementary material for: Neglected tropical diseases in children: An assessment of gaps in research prioritization
Source: PLoS Negl Trop Dis. 2019 Jan 29;13(1):e0007111. doi: 10.1371/journal.pntd.0007111 (PMC6368333; doi:10.1371/journal.pntd.0007111)
Supplement: S3 Appendix — (DOCX) [file pntd.0007111.s004.docx]

| **Neglected Tropical Disease** | **WHO Site URL for Treatment** |
| --- | --- |
| Buruli ulcer | <http://apps.who.int/iris/bitstream/10665/77771/1/9789241503402_eng.pdf?ua=1> |
| Chagas | <http://www.who.int/mediacentre/factsheets/fs340/en/> |
| Dengue | <http://www.who.int/tdr/publications/documents/dengue-diagnosis.pdf> |
| Chikungunya | <http://www.who.int/mediacentre/factsheets/fs327/en/> |
| Dracunculiasis | <http://www.who.int/mediacentre/factsheets/fs359/en/> |
| Echinococcosis | <http://www.who.int/mediacentre/factsheets/fs377/en/> |
| Foodborne Trematodiases | <http://www.who.int/mediacentre/factsheets/fs368/en/> |
| Trypanosomiasis | <http://www.who.int/mediacentre/factsheets/fs259/en/> |
| Leishmaniasis | <http://apps.who.int/iris/bitstream/10665/44412/1/WHO_TRS_949_eng.pdf> |
| Leprosy | <http://apps.who.int/medicinedocs/en/d/Jh2988e/5.html#Jh2988e.5> |
| Lymphatic Filariasis | <http://www.who.int/lymphatic_filariasis/epidemiology/treatment_prevention/en/> |
| Chromoblasto-mycoses | Queiroz-Telles F, Esterre P, Perez-Blanco M, Vitale RG, Salgado CG, Bonifaz A. Chromoblastomycosis: an overview of clinical manifestations, diagnosis and treatment. Med Mycol. 2009 Feb;47(1):3-15. |
| Scabies | <http://www.who.int/lymphatic_filariasis/epidemiology/scabies/en/> |
| Schistosomiasis | <http://www.who.int/mediacentre/factsheets/fs115/en/> |
| Intestinal Nematode Infections | <http://apps.who.int/medicinedocs/en/d/Jh2922e/3.2.html> |
| Taeniasis/  Cysticercosis | <http://www.who.int/mediacentre/factsheets/fs376/en/> |
| Trachoma | <http://www.who.int/mediacentre/factsheets/fs382/en/> |
| Yaws | <http://www.who.int/mediacentre/factsheets/fs316/en/> |
