# Supplementary material for: Neglected tropical diseases in children: An assessment of gaps in research prioritization
Source: PLoS Negl Trop Dis. 2019 Jan 29;13(1):e0007111. doi: 10.1371/journal.pntd.0007111 (PMC6368333; doi:10.1371/journal.pntd.0007111)
Supplement: S1 Table — (DOCX) [file pntd.0007111.s005.docx]

| **Condition** | **Trial Number** | **Title** | **Pediatric Trial** |
| --- | --- | --- | --- |
| Buruli Ulcer | ISRCTN72102977 | Efficacy and host-pathogen response of heat treatment in patients with Buruli ulcer (BU) using a Phase Change Material (PCM) device |  |
| Buruli Ulcer | NCT00321178 | BURULICO Drug Trial Study Protocol: RCT SR8/SR4+CR4, GHANA |  |
| Buruli Ulcer | NCT01659437 | WHO Drug Study for Buruli Ulcer - Comparison of SR8 and CR8 |  |
| Chagas | NCT01549236 | Population Pharmacokinetics Study of Benznidazole in Children With Chagas' Disease | Yes |
| Chagas | NCT01489228 | Proof-of-Concept Study of E1224 to Treat Adult Patients With Chagas Disease |  |
| Chagas | NCT01678599 | Optimization of PCR Technique to Assess Parasitological Response for Patients With Chronic Chagas Disease |  |
| Chagas | NCT02498782 | Study to Evaluate Fexinidazole Dosing Regimens for the Treatment of Adult Patients With Chagas Disease |  |
| Chagas | NCT01566617 | Impact of the Pharmaceutical Care on the Quality of Life in Patients With Chagas Heart Disease |  |
| Chagas | NCT02369978 | CHICAMOCHA 3 - Equivalence of Usual Interventions for Trypanosomiasis (EQUITY) |  |
| Chagas | NCT00875173 | Selenium Treatment and Chagasic Cardiopathy (STCC) |  |
| Chagas | NCT01722942 | Amiodarone Against ICD Therapy in Chagas Cardiomyopathy for Primary Prevention of Death |  |
| Chagas | EUCTR2009-017467-42-ES | Phase II Clinical Trial, Randomized and Open for Etiological Treatment of Chronic Chagas Disease with Posaconazole and Benznidazole |  |
| Chagas | EUCTR2013-000161-36-ES | Phase 2 Study of Posaconazole in Chagas Disease |  |
| Chagas | RBR-6tnw2b | Evaluation of G-CSF treatment of patients with chagasic cardiomyopathy. |  |
| Chagas | RBR-95jnqp | Effect of carvedilol medication and vitamins E and C in patients with Chagas' disease. |  |
| Chagas | NCT02154269 | Evaluation of G-CSF (Colony Stimulating Factor) in Patients With Chronic Chagas Cardiomyopathy |  |
| Chagas | NCT01377480 | A Study of the Use of Oral Posaconazole (POS) in the Treatment of Asymptomatic Chronic Chagas Disease (P05267) |  |
| Chagas | NCT01162967 | Clinical Trial For The Treatment Of Chronic Chagas Disease With Posaconazole And Benznidazole |  |
| Chagas | NCT01755403 | Population Pharmacokinetics in Benznidazole-treated Adults With Chronic With Chagas Disease |  |
| Chagas | NCT00699387 | Population Pharmacokinetics of Benznidazole in Children With Chagas Disease |  |
| Chagas | EUCTR2016-003789-21-ES | Phase II trial for assessing different benznidazol regimens in the treatment of Chagas disease in adult patients on chronic phase . BERINECE project |  |
| Chagas | EUCTR2011-002022-41-ES | Assessment of Therapeutic Response to Benznidazole in patients with Chronic Chagas Disease. |  |
| Chagas | NCT02625974 | Prospective Study of a Pediatric Nifurtimox Formulation for Chagas' Disease | Yes |
| Chagas | NCT03378661 | BENDITA Benznidazole New Doses Improved Treatment and Associations |  |
| Dengue and Chikungunya | NCT00966628 | Effect of Hypertonic Sodium Lactate on sVCAM-1 Level as Surrogate Marker of Endothelial Capillary Leakage in Pediatric Dengue Shock Syndrome Patients (DSS) | Yes |
| Dengue and Chikungunya | NCT02230163 | Clinical Evaluation of Anti-CHIKV Hyperimmune Intravenous Immunoglobulins | Yes |
| Dengue and Chikungunya | NCT02045069 | Efficacy and Safety of Ivermectin Against Dengue Infection |  |
| Dengue and Chikungunya | NCT01973855 | Chinese and Western Medicine Treatment of Fever Associated With Bleeding Symptoms |  |
| Dengue and Chikungunya | NCT00849602 | The Effect of Chloroquine in the Treatment of Patients With Dengue |  |
| Dengue and Chikungunya | NCT02673840 | Ketotifen as a Treatment for Vascular Leakage During Dengue Fever |  |
| Dengue and Chikungunya | NCT00391313 | CuraChik: A Trial of the Efficacy and Safety of Chloroquine as Therapeutic Treatment of Chikungunya Disease |  |
| Dengue and Chikungunya | TCTR20151110001 | A randomized controlled trial of zinc supplementation for regulation severity of dengue viral infection in children | Yes |
| Dengue and Chikungunya | NCT02406729 | Phase III Trial to Evaluate Efficacy and Safety of a Tetravalent Dengue Vaccine |  |
| Dengue and Chikungunya | SLCTR/2014/023 | Use of Rupatadine in the treatment of acute dengue infection |  |
| Dengue and Chikungunya | NCT02421367 | Study of Varying Injection Schedules of TDENV-PIV Vaccine With AS03B Adjuvant and Placebo in Healthy US Adults |  |
| Dengue and Chikungunya | ChiCTR-IPR-16009233 | Lianbizhi injection for treatment of dengue fever - randomized controlled multicenter clinical study |  |
| Dengue and Chikungunya | SLCTR/2016/019 | Phase III, Double-Blind, Randomized, Placebo-Controlled Trial to Investigate the Efficacy, Safety and Immunogenicity of a Tetravalent Dengue Vaccine (TDV) Administered Subcutaneously in Healthy Children Aged 4-16 Years Old | Yes |
| Dengue and Chikungunya | CTRI/2016/09/007303 | Role of Homoeopathy in dengue prevention |  |
| Dengue and Chikungunya | CTRI/2015/05/005806 | A life saving measure for Dengue patients: Carica Papaya Leaf Extract |  |
| Dengue and Chikungunya | ChiCTR-IPR-15006778 | A randomized controlled clinical trial of Tanreqing Injection in patients with dengue fever. |  |
| Dengue and Chikungunya | CTRI/2014/10/005120 | A clinical trial to study the mechanism of action of papaya leaf extract in dengue patients |  |
| Dengue and Chikungunya | EUCTR2014-001534-29-Outside-EU/EEA | Study of Sanofi Pasteur's dengue vaccine in Subjects Aged 2 to 45 Years in the Philippines |  |
| Dengue and Chikungunya | NCT00842530 | Efficacy and Safety of Dengue Vaccine in Healthy Children | Yes |
| Dengue and Chikungunya | NCT01436396 | Study of Yellow Fever Vaccine Administered With Tetravalent Dengue Vaccine in Healthy Toddlers | Yes |
| Dengue and Chikungunya | NCT01411241 | Study of a Booster Injection of Pentaxim^TM^ Vaccine Administered With Dengue Vaccine in Healthy Toddlers | Yes |
| Dengue and Chikungunya | NCT00993447 | Immunogenicity and Safety of Sanofi Pasteur's CYD Dengue Vaccine in Healthy Children and Adolescents in Latin America | Yes |
| Dengue and Chikungunya | NCT00788151 | Study of Chimerivax^TM^ Tetravalent Dengue Vaccine in Healthy Peruvian Children Aged 2 to 11 Years | Yes |
| Dengue and Chikungunya | NCT01254422 | Study of a Tetravalent Dengue Vaccine in Healthy Children Aged 2 to 11 Years in Malaysia | Yes |
| Dengue and Chikungunya | ISRCTN39575233 | A randomized, placebo-controlled, partially blinded (drug versus placebo) trial of early corticosteroid therapy in Vietnamese children and young adults with suspected dengue infection | Yes |
| Dengue and Chikungunya | NCT01064141 | A Study of Dengue Vaccine in Healthy Toddlers Aged 12 to 15 Months in the Philippines | Yes |
| Dengue and Chikungunya | NCT01187433 | Study of CYD Dengue Vaccine in Healthy Children and Adolescents in South America | Yes |
| Dengue and Chikungunya | NCT00880893 | Study of Sanofi Pasteur's CYD Dengue Vaccine in Healthy Subjects in Singapore |  |
| Dengue and Chikungunya | NCT02193087 | Safety and Immunogenicity of Three Formulations of Takeda's Tetravalent Dengue Vaccine Candidate (TDV) |  |
| Dengue and Chikungunya | NCT01134263 | Study of a Tetravalent Dengue Vaccine in Healthy Adults in Australia |  |
| Dengue and Chikungunya | NCT00468858 | A Study of Two Doses of WRAIR Dengue Vaccine Administered Six Months Apart to Healthy Adults and Children |  |
| Dengue and Chikungunya | NCT01488890 | Immune Response to Different Schedules of a Tetravalent Dengue Vaccine Given With or Without Yellow Fever Vaccine |  |
| Dengue and Chikungunya | NCT01030211 | Adult Dengue Platelet Study |  |
| Dengue and Chikungunya | NCT01511250 | Study to Investigate the Safety and Immunogenicity of a Tetravalent Chimeric Dengue Vaccine in Healthy Volunteers Between the Ages of 1.5-45 Years |  |
| Dengue and Chikungunya | NCT02425098 | Safety and Immunogenicity With Two Different Serotype 2 Potencies of Tetravalent Dengue Vaccine (TDV) in Adults in Singapore |  |
| Dengue and Chikungunya | NCT00617344 | Immunogenicity and Safety of Three Formulations of Dengue Vaccines in Healthy Adults Aged 18 to 45 Years in the US |  |
| Dengue and Chikungunya | NCT01550289 | Study of a Tetravalent Dengue Vaccine in Healthy Adult Subjects Aged 18 to 45 Years in India |  |
| Dengue and Chikungunya | NCT00875524 | Study of Chimerivax^TM^ Tetravalent Dengue Vaccine in Healthy Subjects |  |
| Dengue and Chikungunya | NCT00740155 | Safety and Immunogenicity of Formulations of Dengue Vaccines in Healthy Flavivirus-Naïve Adults |  |
| Dengue and Chikungunya | NCT00239577 | A Trial of a Walter Reed Army Institute of Research (WRAIR) Live Attenuated Virus Tetravalent Dengue Vaccine in Healthy US Adults |  |
| Dengue and Chikungunya | NCT00370682 | A Phase II Trial of a Live Attenuated Virus Tetravalent Dengue Vaccine in Healthy Adults in Thailand |  |
| Dengue and Chikungunya | NCT00350337 | A Phase II Trial of a WRAIR Live Attenuated Virus Tetravalent Dengue Vaccine in Healthy US Adults |  |
| Dengue and Chikungunya | NCT01619969 | Celgosivir as a Treatment Against Dengue |  |
| Dengue and Chikungunya | NCT01443247 | Role of Andi-d in Dengue Fever: a Pilot Study |  |
| Dengue and Chikungunya | EUCTR2006-006165-17-FR | Efficacy of an early administrationof L0013CP 10 mg/day versus placebo during 4 weeks in the treatment of infectious diseases induced arthritis painful symptoms. A multicenter, randomised, double bling, placebo controlled clinical trial in adult patient suffering from chikungunya fever. |  |
| Dengue and Chikungunya | EUCTR2015-004037-26-AT | Clinical trial to investigate the efficacy, safety and tolerability of MV-CHIK, a novel vaccine that prevents infection with Chikungunya Virus |  |
| Dengue and Chikungunya | NCT01374516 | Study of a Novel Tetravalent Dengue Vaccine in Healthy Children and Adolescents Aged 9 to 16 Years in Latin America | Yes |
| Dengue and Chikungunya | NCT02747927 | Efficacy, Safety and Immunogenicity of Takeda's Tetravalent Dengue Vaccine (TDV) in Healthy Children | Yes |
| Dengue and Chikungunya | NCT01373281 | Study of a Novel Tetravalent Dengue Vaccine in Healthy Children Aged 2 to 14 Years in Asia | Yes |
| Dengue and Chikungunya | NCT02302066 | Safety and Immunogenicity of Different Schedules of Takeda's Tetravalent Dengue Vaccine Candidate (TDV) in Healthy Participants | Yes |
| Dengue and Chikungunya | NCT02993757 | Immunogenicity and Safety of a Tetravalent Dengue Vaccine Administered Concomitantly or Sequentially With Gardasil | Yes |
| Dengue and Chikungunya | NCT02979535 | Immunogenicity and Safety of a Tetravalent Dengue Vaccine Administered Concomitantly or Sequentially With Cervarix | Yes |
| Dengue and Chikungunya | NCT02623725 | Study of a Booster Dose of a Tetravalent Dengue Vaccine in Subjects Who Previously Completed the 3-dose Schedule | Yes |
| Dengue and Chikungunya | NCT02628444 | Immunogenicity and Safety of 3-Dose and Booster Dose of Tetravalent Dengue Vaccine in Healthy Subjects 9 to 50 Years of Age |  |
| Dengue and Chikungunya | NCT02992418 | Study of a Tetravalent Dengue Vaccine Administered Concomitantly or Sequentially With Adacel in Healthy Subjects |  |
| Dengue and Chikungunya | NCT02562482 | Trial for Safety and Immunogenicity of a Chikungunya Vaccine, VRC-CHKVLP059-00-VP, in Healthy Adults |  |
| Dengue and Chikungunya | NCT01696422 | Phase II Trial to Evaluate Safety and Immunogenicity of a Dengue 1,2,3,4 (Attenuated) Vaccine |  |
| Dengue and Chikungunya | NCT02861586 | Phase II Study to Evaluate Safety and Immunogenicity of a Chikungunya Vaccine |  |
| Dengue and Chikungunya | NCT02824198 | Immunogenicity and Safety of a Tetravalent Dengue Vaccine Booster Injection in Subjects Who Previously Completed a 3-dose Schedule |  |
| Dengue and Chikungunya | NCT02678455 | Safety and Immunogenicity of the Dengue Virus Vaccine TV005 (TetraVax-DV TV005) in Healthy Adults, Adolescents, and Children in Dhaka, Bangladesh |  |
| Dengue and Chikungunya | NCT03058471 | Efficacy of Starting Methotrexate Early in Chikungunya Arthritis |  |
| Dengue and Chikungunya | SLCTR/2015/012 | Effects of Vitamin E Supplementation on the clinical outcome of Dengue Fever and Dengue Haemorrhagic Fever in Children | Yes |
| Dengue and Chikungunya | PER-037-11 | Immunogenicity and Safety of Yellow Fever Vaccine (Stamaril) Administered Concomitantly with Tetravalent Dengue Vaccine in Healthy Toddlers at 12-13 Months of Age in Colombia and Peru | Yes |
| Echinococcosis | ChiCTR-TRC-13003740 | A randomized, multicenter, positive drug controlled clinical trials to compare the 12 consecutive months efficacy and safety of albendazole emulsion and albendazole tablets in the treatment of hepatic cystic echinococcosis. |  |
| Foodborne Trematodiases | IRCT201105291155N13 | Ivermectin effect in treatment of patients with human fascioliasis |  |
| Human African Trypanosomiasis | NCT03087955 | Prospective Study on Efficacy and Safety of SCYX-7158 in Patients Infected by Human African Trypanosomiasis Due to T.b. Gambiense |  |
| Human African Trypanosomiasis | NCT03025789 | Fexinidazole in Human African Trypanosomiasis Due to T.b. Gambiense at Any Stage |  |
| Human African Trypanosomiasis | NCT00906880 | Clinical Study to Assess the Tolerability, Feasibility and Effectiveness of Nifurtimox and Eflornithine (NECT) for the Treatment of Trypanosoma Brucei Gambiense Human African Trypanosomiasis (HAT) in the Meningo-encephalitic Phase |  |
| Human African Trypanosomiasis | NCT02184689 | Efficacy and Safety of Fexinidazole in Children at Least 6 Years Old and Weighing Over 20 kg With Human African Trypanosomiasis (HAT) Due to T.b. Gambiense: a Prospective, Multicentre, Open Study, plug-in to the Pivotal Study | Yes |
| Human African Trypanosomiasis | NCT01685827 | Pivotal Study of Fexinidazole for Human African Trypanosomiasis in Stage 2 |  |
| Human African Trypanosomiasis | NCT02169557 | Efficacy and Safety of Fexinidazole in Patients With Stage 1 or Early Stage 2 Human African Trypanosomiasis (HAT) Due to T.b. Gambiense: a Prospective, Multicentre, Open-label Cohort Study, plug-in to the Pivotal Study |  |
| Leishmaniasis | NCT00487253 | Oral Miltefosine for the Treatment of Pediatric Cutaneous Leishmaniasis in Colombia | Yes |
| Leishmaniasis | NCT01566552 | Single Dose Liposomal Amphotericin B for Visceral Leishmaniasis |  |
| Leishmaniasis | NCT00840359 | Study of the Efficacy of Daylight Activated Photodynamic Therapy in the Treatment of Cutaneous Leishmaniasis |  |
| Leishmaniasis | NCT01138956 | Immune Response of Visceral Leishmaniasis PatientsTreated With Antimonial Plus N-Acetylcysteine |  |
| Leishmaniasis | NCT01310738 | Efficacy and Safety Study of Drugs for Treatment of Visceral Leishmaniasis in Brazil |  |
| Leishmaniasis | NCT00317629 | Controlled Nitric Oxide Releasing Patch Versus Meglumine Antimoniate in the Treatment of Cutaneous Leishmaniasis |  |
| Leishmaniasis | NCT00832208 | Open-Label, Sequential Step, Safety and Efficacy Study to Determine the Optimal Single Dose of Ambisome for Patients With VL |  |
| Leishmaniasis | NCT01953744 | High Dose Fluconazole in Cutaneous Leishmaniasis in Bahia and Manaus |  |
| Leishmaniasis | NCT01494350 | WR 279,396 Open Label Treatment Protocol in Tunisia |  |
| Leishmaniasis | NCT00682656 | Safety and Efficacy of Azithromycin to Treat Cutaneous Leishmaniasis |  |
| Leishmaniasis | NCT01380301 | Treatment of Cutaneous Leishmaniasis With a Combination of Miltefosine and Antimony |  |
| Leishmaniasis | NCT01980199 | Trial to Determine Efficacy of Fexinidazole in Visceral Leihmaniasis Patients in Sudan |  |
| Leishmaniasis | NCT01140191 | Safety, Efficacy, and PK of Topical Paromomycin/Gentamicin Cream for Treatment of Cutaneous Leishmaniasis |  |
| Leishmaniasis | NCT02193022 | Miltefosine for Children With PKDL | Yes |
| Leishmaniasis | NCT02687971 | Thermotherapy + a Short Course of Miltefosine for the Treatment of Uncomplicated Cutaneous Leishmaniasis in the New WorldÂ¨ |  |
| Leishmaniasis | NCT01301937 | Low Antimonial Dosage in American Mucosal Leishmaniasis |  |
| Leishmaniasis | NCT02530697 | The Association of Miltefosine and Pentoxifylline to Treat Mucosal Leishmaniasis: A Clinical Trial in Brazil |  |
| Leishmaniasis | NCT02894008 | A Study of a New Leishmania Vaccine Candidate ChAd63-KH |  |
| Leishmaniasis | IRCT138903023862N3 | Comparing two methods of oral Azithromycin and cryotherapy in cutaneous leishmaniasis treatment in children | Yes |
| Leishmaniasis | CTRI/2009/091/000058 | Comparing the treatments of Two Different Lipid preparations of Amphotericin B for the treatment of Kala Azar |  |
| Leishmaniasis | CTRI/2012/08/002891 | An open-label, prospective, non randomised, non comparative, multicenter, observational pharmacovigilence study of the safety and effectiveness of new treatment modalities to treat VL in public sector of India. |  |
| Leishmaniasis | CTRI/2009/091/000318 | A clinical trial to study the effects of various combinations for the treatment of Kala-Azar |  |
| Leishmaniasis | ACTRN12612000367842 | Feasibility of using single dose liposomal Amphotericin B for the treatment of Visceral Leishmaniasis in Bangladesh |  |
| Leishmaniasis | IRCT2013092414746N1 | Treatment of patients with ZCL |  |
| Leishmaniasis | ACTRN12614001288617 | A clinical trial to assess the safety and effect of heat therapy in comparison to standard intra-lesional sodium stibogluconate for cutaneous leishmaniasis |  |
| Leishmaniasis | IRCT138903063862N4 | Comparing improvement efficacy of leishmaniasis patients with three treatment methods including combined cryotherapy and interalesional meglumine antimoniate (Glucantime) vs. cryotherapy and interalesional glucantime alone for the treatment of cutaneous leishmaniasis |  |
| Leishmaniasis | IRCT201108301475N6 | Double blind, randomized phase 3 efficacy trial of topical nano-liposomal paromomycin in the treatment of cutaneous leishmaniasis caused by L. major |  |
| Leishmaniasis | IRCT201101313862N6 | Comparing improvement of lesions in leishmaniasis patients with two treatment methods including trichloroacetic Acid (TCA) and interalesional meglumine antimoniate (Glucantime) for the treatment of Lupoid leishmaniasis |  |
| Leishmaniasis | IRCT138802131871N1 | Comparing efficacy of two treatment methods including topical liposomal Amphotericin B versus intralesional Meglumine Antimoniate (Glucantime) in the Treatment of Cutaneous Leishmaniasis |  |
| Leishmaniasis | IRCT138904091159N7 | Comparison between two groups in the treatment of cutaneous leishmaniasis |  |
| Leishmaniasis | IRCT138710191535N1 | Evaluation of efficacy of topical nitric oxide in treatment of cutaneous leishmaniasis |  |
| Leishmaniasis | IRCT138804162082N1 | Comparing theraputic response to systemic Meglumine Antimoniate (Glucantime) in children with acute Cutaneous Leishmaniasis versus adult patients |  |
| Leishmaniasis | IRCT138710221542N1 | Comparison of two regimens of fluconazol in treatment of cutaneous lieshmaniasis |  |
| Leishmaniasis | IRCT138901312082N2 | Comparing efficacy of two treatment methods including topical liposomal Azithromycin versus intralesional Meglumine Antimoniate (Glucantime) in the treatment of cutaneous Leishmaniasis |  |
| Leishmaniasis | IRCT2014031216955N1 | Topical treatment of cutaneous leishmaniasis with lipozomal azithromycin |  |
| Leishmaniasis | IRCT2015082523753N1 | Effectiveness of traditional medicine products Juniperus excelsa M.Bieb in the treatment of cutaneous leishmaniasis |  |
| Leishmaniasis | JPRN-UMIN000010994 | Double blinded randomised placebo controlled phase II trial of Shiunko Ointment local application twice a day for 4 weeks in Ethiopian patients with localized cutaneous Leishmaniasis. |  |
| Leishmaniasis | ACTRN12609001029280 | Liposomal amphotericin B for the treatment of Bangladeshi patients with visceral leishmaniasis. |  |
| Leishmaniasis | IRCT138903183862N5 | Evaluating the effects of thermotherapy on cutaneous leishmaniasis |  |
| Leishmaniasis | ACTRN12610000130066 | Liposomal amphotericin B for the treatment of Nepalese patients with visceral leishmaniasis. |  |
| Leishmaniasis | CTRI/2011/11/002145 | A Prospective Study to Assess Safety of Single-Dose Regimen at Two Dose Levels of FUNGISOME^TM^ in Treatment of Visceral Leishmaniasis (Kala-Azar) |  |
| Leishmaniasis | NCT01032187 | Amphotericin B to Treat Visceral Leishmaniasis in Brazilian Children | Yes |
| Leishmaniasis | NCT02431143 | Pharmacokinetics/Safety of Miltefosine Allometric Dose for the Treatment of Visceral Leishmaniasis in Children in Eastern Africa | Yes |
| Leishmaniasis | NCT00604955 | Expand Access/Assess Safety and Efficacy of Paromomycin IM Injection for the Treatment of Visceral Leishmaniasis |  |
| Leishmaniasis | NCT00696969 | Safety and Efficacy Study to Evaluate Different Combination Treatment Regimens for Visceral Leishmaniasis |  |
| Leishmaniasis | NCT00523965 | Combination Therapy in Indian Visceral Leishmaniasis |  |
| Leishmaniasis | NCT01122771 | Phase III, Study of Three Short Course Combo (Ambisome, Miltefosine, Paromomycin) Compared With AmBisome for the Treatment of VL in Bangladesh |  |
| Leishmaniasis | NCT00876824 | To Study the Effect Of Single Infusions Of Amphotericin B Lipid Preparations in Treatment of Patients Of Kala Azar |  |
| Leishmaniasis | NCT00471705 | Efficacy and Safety of Miltefosine or Thermotherapy for Cutaneous Leishmaniasis in Colombia. |  |
| Leishmaniasis | NCT00628719 | Single Infusion of Liposomal Amphotericin B in Indian Visceral Leishmaniasis |  |
| Leishmaniasis | NCT01790659 | Phase 3 Study of Walter Reed (WR) 279,396 and Paromomycin Alone for the Treatment of Cutaneous Leishmaniasis in Panama |  |
| Leishmaniasis | NCT00606580 | Phase 3 Study to Evaluate WR 279,396 vs. Paromomycin Alone to Treat Cutaneous Leishmaniasis (in Tunisia) |  |
| Leishmaniasis | NCT00629031 | An Open Lable Randomised Study to Assess the Safety and Efficacy of Short Course Paromomycin in Visceral Leishmaniasis |  |
| Leishmaniasis | NCT03311607 | Safety and Effectiveness of Short-course AmBisome in the Treatment of PKDL in Bangladesh |  |
| Leishmaniasis | NCT00317980 | Safety and Efficacy of Low-Dose Pentavalent Antimony for Treatment of Cutaneous Leishmaniasis |  |
| Leishmaniasis | NCT01069198 | A Community Trial for Visceral Leishmaniasis (VL) |  |
| Leishmaniasis | NCT00370825 | Combination Chemotherapy for the Treatment of Indian Kala-Azar |  |
| Leishmaniasis | NCT00600548 | Trial of Miltefosine in Cutaneous Leishmaniasis (Brazil) |  |
| Leishmaniasis | NCT01381055 | Antimony Plus Pentoxifylline in Cutaneous Leishmaniasis |  |
| Leishmaniasis | NCT02919605 | Efficacy and Safety of Pentamidine (7mg/kg) for Patients With Cutaneous Leishmaniasis Caused by L. Guyanensis |  |
| Leishmaniasis | NCT01067443 | Clinical Trial to Assess the Safety and Efficacy of Sodium Stibogluconate (SSG) and AmBisome Combination, Miltefosine and AmBisome and Miltefosine Alone for the Treatment Visceral Leishmaniasis in Eastern Africa |  |
| Leishmaniasis | NCT00371995 | Short Course of Miltefosine and Liposomal Amphotericin B for Kala-azar |  |
| Leishmaniasis | NCT00429715 | Safety/Efficacy Trial of Killed Leishmania Vaccine in Volunteers With no Response to Leishmanin |  |
| Leishmaniasis | NCT00351520 | Efficacy Trial on Oral Miltefosine in Comparison With Glucantime in the Treatment of ACL Caused by L. Tropica |  |
| Leishmaniasis | NCT01328457 | An Effectiveness Study of Paromomycin IM Injection (PMIM) for the Treatment of Visceral Leishmaniasis (VL) in Bangladesh |  |
| Leishmaniasis | NCT00490230 | Cosmetic Outcome of Leishmaniasis Scar After WR279396 Application |  |
| Leishmaniasis | NCT01661296 | Efficacy of Radio-frequency Induced Heat (RFH)Therapy in Treatment of Cutaneous Leishmaniasis in India |  |
| Leishmaniasis | NCT00429780 | Safety/Efficacy Trial of Killed Leishmania Vaccine in Volunteers With Positive Response to Leishmanin (LST>0) |  |
| Leishmaniasis | NCT01300975 | Intralesional Antimony for Bolivian Cutaneous Leishmaniasis |  |
| Leishmaniasis | NCT00469495 | Antihelminthic Therapy Combined With Antimony in the Treatment of Cutaneous Leishmaniasis |  |
| Leishmaniasis | NCT01845727 | Topical 3% Amphotericin B Cream for the Treatment of Cutaneous Leishmaniasis in Colombia |  |
| Leishmaniasis | NCT01464242 | Add-on Study of Pentoxifylline in Cutaneous Leishmaniasis |  |
| Leishmaniasis | NCT01360762 | Prophylaxis of Visceral Leishmaniasis Relapses in HIV Co-infected Patients With Pentamidine: a Cohort Study |  |
| Leishmaniasis | NCT00996463 | Rationales for Wound Care Management in Old World Cutaneous Leishmaniasis Patients |  |
| Leishmaniasis | NCT00381394 | A Study Evaluating Sitamaquine Compared With Amphotericin B In The Treatment Of Visceral Leishmaniasis. |  |
| Leishmaniasis | NCT01380314 | Oral Miltefosine Plus Topical Imiquimod to Treat Cutaneous Leishmaniasis |  |
| Leishmaniasis | NCT01462500 | Pharmacokinetics of Miltefosine in Children and Adults |  |
| Leishmaniasis | NCT00497601 | A Phase II Study To Assess Safety and Efficacy Of Short-Course Regimens Of Amphotericin B Emulsion In Kala-Azar |  |
| Leishmaniasis | NCT03294161 | Fourth-generation Immucillin Derivative DI4G Associated Therapy in Cutaneous Leishmaniasis |  |
| Leishmaniasis | NCT01011309 | A Study of the Efficacy and Safety of the LEISH-F2 + MPL-SE Vaccine for Treatment of Cutaneous Leishmaniasis |  |
| Leishmaniasis | NCT01377974 | Clinical Trial of Miltefosine to Treat Mucosal Leishmaniasis |  |
| Leishmaniasis | NCT01635777 | Safety and Efficacy of Oral Miltefosine in Patients With Post Kala Azar Dermal Leishmaniasis (PKDL) |  |
| Leishmaniasis | NCT01975051 | A Study to Explore Association of Treatment Regimens for Visceral Leishmaniasis, Host Immunological, Genetical and Nutrition Factors With Post-kala-azar Dermal Leishmaniasis (PKDL) |  |
| Leishmaniasis | NCT01083576 | Pharmacokinetics, Safety, and Efficacy Trial of WR 279,396 (Paromomycin + Gentamicin Topical Cream) and Paromomycin Topical Cream for the Treatment of Cutaneous Leishmaniasis in Panama |  |
| Leishmaniasis | NCT01032382 | Safety, Efficacy and Pharmacokinetics (PK) Study of WR 279,396 Versus Paromomycin for Treatment of Cutaneous Leishmaniasis (Peru-PK) |  |
| Leishmaniasis | NCT02025491 | Liposomal Amphotericin in Disseminated Leishmaniasis |  |
| Leishmaniasis | NCT01988909 | WR 279,396 for the Treatment of Cutaneous Leishmaniasis |  |
| Leishmaniasis | NCT00818818 | Low-dose Pentavalent Antimony Treatment of Cutaneous Leishmaniasis in Old Age Patients |  |
| Leishmaniasis | NCT01050907 | Miltefosine to Treat Mucocutaneous Leishmaniasis |  |
| Leishmaniasis | NCT01301924 | Comparison of Standard and Alternative Antimonial Dosage in Patients With American Cutaneous Leishmaniasis |  |
| Leishmaniasis | NCT02011958 | Efficacy Trial of Ambisome Given Alone and Ambisome Given in Combination With Miltefosine for the Treatment of VL HIV Positive Ethiopian Patients. |  |
| Leishmaniasis | NCT03009422 | Fractional CO2 Laser With Topical Pentostam Treatment for Cutaneous Leishmaniasis. |  |
| Leprosy | NCT00406861 | Montelukast in ENL Reaction |  |
| Leprosy | NCT02550080 | Clinical Utility Of Genetic Screening For HLA-B*1301, On Susceptibility To Dapsone Hypersensitivity Syndrome |  |
| Leprosy | CTRI/2011/09/002022 | Treatment of Early Neuropathy in Leprosy |  |
| Leprosy | CTRI/2013/05/003607 | Comparison of usefullness and safety of two drugs, Pentoxyphylline and Clofazimine, along with Prednisolone in Leprosy reaction |  |
| Leprosy | JPRN-JMA-IIA00116 | Hansens disease neurogenic pain treated by tramset study |  |
| Leprosy | CTRI/2012/07/002791 | Preventing leprosy by vaccine |  |
| Leprosy | NTR3087 | The combined effect of a single dose of rifampicin and vaccination with BCG, in the prevention of leprosy in contacts of newly diagnosed cases: A randomized controlled trial. |  |
| Leprosy | CTRI/2012/05/002696 | Uniform treatment regimen for all types of leprosy patients |  |
| Leprosy | CTRI/2012/05/002645 | Simpler treatment regimen for patients with few skin patches of leprosy. |  |
| Leprosy | CTRI/2016/12/007558 | Azathioprine in Type 1 Leprosy Reactions-The Leprosy Mission Trusy India |  |
| Leprosy | ISRCTN31894035 | A phase II trial to investigate the safety of early high dose methylprednisolone in acute leprous neuritis and leprosy type 1 reactions with neuritis in Nepal |  |
| Leprosy | NCT00669643 | Uniform Multidrug Therapy Regimen for Leprosy Patients |  |
| Leprosy | NCT01290744 | Effect of Additional Clofazimine on Erythema Nodosum Leprosum (ENL) Reactions in Leprosy |  |
| Leprosy | NCT00919815 | Ciclosporin in the Management of New Type 1 Reactions in Leprosy |  |
| Leprosy | NCT00860717 | The Use of Low Level Laser Therapy for Wound Healing in Leprosy Patients |  |
| Leprosy | NCT00919776 | Ciclosporin in the Management of Chronic or Recurrent Erythema Nodosum Leprosum |  |
| Leprosy | NCT00919451 | Ciclosporin in the Management of Steroid Resistant Type 1 Reactions in Leprosy |  |
| Leprosy | NCT00919542 | Ciclosporin in the Management of New Erythema Nodosum Leprosum |  |
| Lymphatic Filariasis | CTRI/2011/12/002268 | Management of Lymphatic Filariasis with Homeopathic Medicines. |  |
| Lymphatic Filariasis | CTRI/2016/10/007399 | Triple drug study for lymphatic filariasis elimination |  |
| Lymphatic Filariasis | CTRI/2012/03/002539 | Morbidity reduction of Lymphatic Filariasis using integrative treatment as self care &amp; home based procedure |  |
| Lymphatic Filariasis | CTRI/2014/02/004375 | Low dose DEC as an alternative to current dose used in Lymphatic Filariasis program |  |
| Lymphatic Filariasis | CTRI/2012/02/002467 | A clinical trial to study the effectiveness of combination of two drugs compared to standard single drug treatment in practice for the clearance of filarial infection from the infected individuals |  |
| Lymphatic Filariasis | CTRI/2015/03/005611 | To observe the effect of Unani medicines on certain Biochemical markers like VEGF-A, VEGF-C and inflammatory cytokines in patients of Lymphatic Filariasis (Daul Feel) |  |
| Lymphatic Filariasis | NCT01586169 | Safety of the Co-administration of Three Drugs for Trachoma and Lymphatic Filariasis Elimination |  |
| Lymphatic Filariasis | NCT00375583 | Effect of Albendazole Dose on Clearance of Filarial Worms |  |
| Lymphatic Filariasis | NCT00339417 | Effect of Albendazole Dose on Clearance of Filarial Worms |  |
| Lymphatic Filariasis | NCT01593722 | Post-treatment Effects of Ivermectin (IVM) or Diethylcarbamazine (DEC) in Loiasis |  |
| Lymphatic Filariasis | NCT02005653 | Alternate Chemotherapy Regimens for the Clearance of W.Bancrofti Infection |  |
| Lymphatic Filariasis | NCT00511004 | Effect of Albendazole Dose on Treatment of Lymphatic Filariasis |  |
| Lymphatic Filariasis | NCT01111305 | Reslizumab to Prevent Post-treatment Eosinophilia in Loiasis |  |
| Lymphatic Filariasis | NCT02899936 | Death to Onchocerciasis and Lymphatic Filariasis (DOLF) Triple Drug Therapy for Lymphatic Filariasis |  |
| Lymphatic Filariasis | NCT02974049 | Lymphatic Filariasis (LF) in Ivory Coast |  |
| Lymphatic Filariasis | NCT01975441 | Eval 3-Drug Therapy Diethylcarbamize, Albendazole and Ivermectin That Could Accelerate LF Elimination Outside of Africa |  |
| Mycetoma, Chromoblastomycosis, and Other Deep Mycoses | EUCTR2008-003560-19-ES | Study for safety and efficacy evalution of ciclopirox olamine cream suffering dermatomycosis |  |
| Mycetoma, Chromoblastomycosis, and Other Deep Mycoses | CTRI/2009/091/001048 | A clinical trial to study the effect of a combination product of Eberconazole 1% and Mometasone 0.1% in patients with inflamed cutaneous mycoses. |  |
| Mycetoma, Chromoblastomycosis, and Other Deep Mycoses | CTRI/2010/091/003026 | A Post marketing comparative study of three topical medications, Setaconazole 2%, Terbinafine 1% and Luliconazole 1% in patients with fungal infections of the skin |  |
| Mycetoma, Chromoblastomycosis, and Other Deep Mycoses | EUCTR2010-024212-33-FR | Pharmacocinétique de population des anti-infectieux (Ceftazidime, Ciprofloxacine et Voriconazole). |  |
| Mycetoma, Chromoblastomycosis, and Other Deep Mycoses | NCT01259336 | Efficacy Of Itraconazole In Chronic Cavitary Pulmonary Aspergillosis |  |
| Mycetoma, Chromoblastomycosis, and Other Deep Mycoses | EUCTR2015-003940-38-ES | Study of posaconazole blood levels comparing two different oral presentations, a syrup and a solid tablet, in patients with leukaemia at a high risk of fungal infections |  |
| Onchocerciasis | ISRCTN95189962 | Evaluation of the impact of large scale, community directed delivery of doxycycline for the treatment of onchocerciasis |  |
| Onchocerciasis | PACTR201303000464219 | Onchocerciasis Ocular Pathology post Ivermectin |  |
| Onchocerciasis | PACTR201608001754356 | The efficacy of Rifapentine plus Moxifloxacin against Onchocercia-sis. |  |
| Onchocerciasis | NCT00790998 | Study Comparing Moxidectin And Ivermectin In Subjects With Onchocerca Volvulus Infection |  |
| Onchocerciasis | NCT03238131 | IVM Alone vs ALB + IVM Against Onchocerciasis |  |
| Onchocerciasis | NCT00300768 | Study Evaluating Orally Administered Moxidectin In Subjects With Onchocerca Volvulus Infection |  |
| Onchocerciasis | NCT02899936 | Death to Onchocerciasis and Lymphatic Filariasis (DOLF) Triple Drug Therapy for Lymphatic Filariasis |  |
| Onchocerciasis | NCT02078024 | Efficacy of Ivermectin and Albendazole Against Onchocerciasis in the Volta Region, Ghana |  |
| Rabies | NCT02040090 | Phase II/III Study of the Safety and Effectiveness of HRIG With Co-administration of Active Rabies Vaccine in Healthy Subjects |  |
| Rabies | NCT02490956 | Diagnostic Immunization With Rabies Vaccine in Patients With PID |  |
| Rabies | NCT02276625 | One-visit Multi-site Intradermal Rabies Vaccination - Dose Finding |  |
| Rabies | ChiCTR-PIR-16008129 | A phase III study for freeze-dried rabies human diploid cell vaccine |  |
| Rabies | NCT01137045 | Immunogenicity and Safety Study of A New Chromatographically Purified Vero Cell Rabies Vaccine With ID Regimen and ERIG |  |
| Rabies | NCT02564471 | Effect of Antimalarial Drugs to Rabies Vaccine for Post-exposure Prophylaxis. |  |
| Rabies | NCT01641315 | Immunogenicity Study of a Reduced (4-dose) Vaccine Schedule and Rabies Immunoglobulins |  |
| Rabies | CTRI/2016/08/007137 | Immunogenicity and safety study of Rabies G protein Vaccine administered as a simulated post-exposure immunization in healthy volunteers |  |
| Rabies | CTRI/2016/01/006477 | A Post Marketing Surveillance Study to Evaluate the Safety of Abhayrab Vaccine Reconstituted to 1 mL Administered Intradermally in Category II Animal Exposure Subjects in India |  |
| Rabies | NCT01930357 | Purified Vero Rabies Vaccine-Serum Free Compared to Human Diploid Cell Vaccine in a Pre-exposure Prophylaxis Regimen | Yes |
| Rabies | NCT02991872 | A Study to Evaluate Persistence of Immune Responses After Post-exposure Prophylaxis of Rabipur (Purified Chicken-embryo Cell Rabies Vaccine) in Chinese Children | Yes |
| Rabies | NCT00708084 | Randomized Phase II Trial on Safety and Neutralizing Activity of CL184 and Rabies Vaccine Versus Human Rabies Immune Globulin (HRIG) and Rabies Vaccine in Children and Adolescents | Yes |
| Rabies | NCT01821911 | The Post-Marketing Safety and Immunogenicity Research of Speeda Rabies Vaccine for Human Use |  |
| Rabies | NCT02288286 | Safety and Immunogenicity of Freeze-dried Rabies Vaccine (MRC-5 Cell) in Chinese Humans |  |
| Rabies | NCT02491541 | A Phase 3 Clinical Trial for a Rabies Vaccine (Vero Cell) for Human Use in Healthy Chinese Subjects |  |
| Rabies | NCT02177032 | Safety and Immunogenicity of Two Intradermal Rabies Vaccine Regimens Administered With and Without Human Rabies Immunoglobulin in Subjects > 1 Years of Age |  |
| Rabies | NCT00825305 | Safety and Immunogenicity (Non-inferiority) of a Purified Chick Embryo Cell Vaccine Vaccine Administered in Two Different Schedules (Conventional Versus Abbreviated Schedule) |  |
| Rabies | NCT01339312 | Study of the Purified Vero Rabies Vaccine - Serum Free in Comparison With the Reference Purified Vero Rabies Vaccine |  |
| Rabies | NCT01662440 | Safety and Immunogenicity of 2 Different Vaccination Schedules of Rabies and Japanese Encephalitis Vaccines in Healthy Adult Subjects |  |
| Rabies | NCT01680016 | A Randomized, Open-label Study Comparing Two Different Rabies Vaccine Schedules in Chinese Children and Older Adults |  |
| Rabies | NCT00345319 | Purified Rabies Vaccine for Human Use (Chick-embryo Cell) |  |
| Rabies | NCT01388985 | Simplifying the Rabies Pre-exposure Vaccination |  |
| Rabies | NCT01784874 | Comparison of Purified Vero Rabies Vaccine, Serum Free With Human Diploid Cell Vaccine in Pre-exposure Use |  |
| Rabies | NCT00948272 | Study of Purified Vero Rabies Vaccine Serum Free Compared With Reference Purified Vero Rabies Vaccine in Healthy Adults |  |
| Rabies | NCT01877395 | Study of Purified Vero Rabies Vaccine and Rabies Human Diploid Cell Vaccine in a Simulated Rabies Post-exposure Regimen |  |
| Rabies | NCT02559921 | Safety and Immunogenicity Study of Recombinant Human Rabies Immunoglobin (rhRIG) in Combination With Rabies Vaccine for Human Use With Human Rabies Immune Globulin (HRIG) in Combination With Rabies Vaccine for Human Use in Healthy Adult Subjects. |  |
| Rabies | NCT01365494 | Immunogenicity and Safety of Rabies Vaccine, Administered With Two Different Simulated Post Exposure Schedules |  |
| Rabies | NCT01228383 | Rabies Virus Neutralizing Activity and Safety of CL184, a Monoclonal Antibody Cocktail, in Simulated Rabies Post-Exposure Prophylaxis in Healthy Adults |  |
| Rabies | NCT02956746 | A Comparison of the Safety, PD and PK of a Single Dose of SYN023 Administered With Licensed Rabies Vaccines |  |
| Rabies | NCT00656097 | A Randomized Phase II Trial to Compare the Safety and Neutralizing Activity of CL184 in Combination With Rabies Vaccine vs. HRIG or Placebo in Combination With Rabies Vaccine in Healthy Adult Subjects |  |
| Rabies | NCT01044199 | Study of Intradermal Administration of PCEC Rabies Vaccine |  |
| Rabies | NCT02956421 | Efficacy and Safety of a PIKA Rabies Vaccine Containing the PIKA Adjuvant With an Accelerated Regimen |  |
| Rabies | NCT02729168 | Safety Study of Rabies Vaccine INDIRAB Five Doses (0.5ml) Post Exposure Administered Intramuscularly |  |
| Rabies | NCT03093545 | Effect of Rabies Immunoglobulin on Immunogenicity of Post-exposure Rabies Vaccination in Obese Patients |  |
| Rabies | NCT02374814 | Immunogenicity of Rabies Vaccine for Pre Exposure |  |
| Rabies | NCT01827917 | The Protection Effect of Speeda Rabies Vaccine for Human Use |  |
| Rabies | NCT01622062 | Immunogenicity and Safety of Verorab in a "One-week" Intradermal Post-exposure Prophylaxis Regimen |  |
| Scabies and Other Ectoparasites | NCT02407782 | Oral Ivermectin Versus Topical Permethrin to Treat Scabies in Children | Yes |
| Scabies and Other Ectoparasites | CTRI/2014/08/004875 | Safety and protection of intramuscular rabies vaccine. |  |
| Scabies and Other Ectoparasites | JPRN-JapicCTI-132344 | KD-357 Phase II/III Study | Yes |
| Scabies and Other Ectoparasites | CTRI/2011/07/001857 | Immunogenicity and safety of Rabies Vaccine, adimistered with two different simulated post exposure regimens |  |
| Scabies and Other Ectoparasites | CTRI/2012/05/002709 | A Comparator-controlled Study of the Safety and Neutralizing Activity of a Human Monoclonal Antibody to Rabies (SII RMAb) Administered in Conjunction with Rabies Vaccine for Post-exposure rabies Prophylaxis |  |
| Scabies and Other Ectoparasites | CTRI/2012/11/003135 | A study of Purified Vero Rabies vaccine for prophylaxis in patients with rabies exposure |  |
| Scabies and Other Ectoparasites | CTRI/2014/04/004538 | Rabies G Protein Vaccine study for immunization against Rabies |  |
| Scabies and Other Ectoparasites | CTRI/2010/091/000055 | A clinical trial to study immunogenicity and safety of Purified Chick Embryo Cell Culture Rabies Vaccine in post-exposure animal bite cases. |  |
| Scabies and Other Ectoparasites | CTRI/2012/12/003230 | Safety and immunogenicity of rabies vaccines in animal bite cases administered using new one week intradermal regimen |  |
| Scabies and Other Ectoparasites | CTRI/2012/06/002720 | Protection and Safety of rabies vaccine when administered intradermally |  |
| Scabies and Other Ectoparasites | JPRN-UMIN000018466 | Rabies post-exposure prophylaxis by purified chik-embryo cell cultured rabies vaccine made in Japan |  |
| Scabies and Other Ectoparasites | CTRI/2010/091/000214 | A Comparative Phase III Clinical Study of Bharat Biotech International Limited Rabies Vaccine INDIRAB reconstituted with 1mL diluent Vs Reference Vaccine, administered Intramuscularly in healthy volunteers as Pre-Exposure treatment. |  |
| Scabies and Other Ectoparasites | CTRI/2012/12/003225 | to evaluate the safety, tolerability and neutralizing activity of Rabimabs against rabies virus in healthy subjects |  |
| Scabies and Other Ectoparasites | CTRI/2015/06/005838 | To evaluate the safety, tolerability and neutralizing activity of Rabimabs against rabies virus in healthy subjects |  |
| Scabies and Other Ectoparasites | NCT02572986 | A Study to Evaluate the Therapeutic Equivalence of Generic Permethrin Cream 5% to Elimite in the Treatment of Scabies |  |
| Scabies and Other Ectoparasites | NCT02978508 | Bio-equivalence Study Comparing Permethrin Cream, 5% With Elimite in Patients With Active Scabies. |  |
| Scabies and Other Ectoparasites | NCT02094716 | A Dose Ranging Vehicle Controlled Study to Determine the Safety and Efficacy of Permethrin Foam, 5% and Permethrin Foam, 4% for the Treatment of Scabies |  |
| Scabies and Other Ectoparasites | EUCTR2015-000382-31-DE | A clinical study to evaluate immune responses to rabies vaccine in adults who received different primary rabies vaccination regimens |  |
| Scabies and Other Ectoparasites | NCT02775617 | Azithromycin - Ivermectin Mass Drug Administration for Skin Disease |  |
| Scabies and Other Ectoparasites | CTRI/2010/091/000509 | A clinical trial to study immunogenicity and safety of Purified Chick Embryo Cell Culture Rabies Vaccine administered intradermally |  |
| Schistosomiasis | NCT01869465 | Evaluation of Strategies for Improved Uptake of Preventive Treatment for Intestinal Schistosomiasis | Yes |
| Schistosomiasis | NCT01459146 | Artemisinin-based Combination Therapy-Intermittent Preventive Treatment (ACT-IPT) Trial Among Schoolchildren in Kassena-Nankana, Ghana | Yes |
| Schistosomiasis | NCT01541631 | A Study of Co-infections of HIV-1 and Schistosoma Mansoni and Its Impact on Praziquantel Treatment Outcomes |  |
| Schistosomiasis | NCT01260012 | Antioxidant Supplements in the Reversal of Schistosomal Peri-portal Fibrosis |  |
| Schistosomiasis | NCT02806232 | An Open Label Dose Finding Safety and Efficacy in Children and Infants Infected With Schistosomiasis (S.Mansoni) | Yes |
| Schistosomiasis | ISRCTN15280205 | Dose-finding and pharmacokinetic studies of praziquantel in children infected with schistosomes | Yes |
| Schistosomiasis | ISRCTN63657086 | Assessment of the safety and efficacy of different drugs and drug combinations in children infected with schistosomes | Yes |
| Schistosomiasis | ISRCTN00393859 | Praziquantel, praziquantel plus mefloquine and praziquantel plus mefloquine-artesunate in the treatment of schistosomiasis |  |
| Schistosomiasis | NCT00510159 | Comparing Praziquantel Versus Artesunate + Sulfamethoxypyrazine/Pyrimethamine for Treating Schistosomiasis | Yes |
| Schistosomiasis | NCT01901484 | Schistosoma Mansoni Morbidity in Children Aged 1-5 Years | Yes |
| Schistosomiasis | NCT01050374 | Safety and Efficacy of Drug Combinations Against Schistosomiasis | Yes |
| Schistosomiasis | NCT01722539 | Impact IPT With Sulfadoxine-pyrimethamine or Sulfadoxine-pyrimethamine Plus Piperaquine in Schoolchildren | Yes |
| Schistosomiasis | NCT01558336 | Schistosoma Haematobium Infections and Praziquantel | Yes |
| Schistosomiasis | NCT02144389 | Arachidonic Acid Treatment Against Schistosomiasis Infection in Children | Yes |
| Schistosomiasis | NCT00870649 | Efficacy of Bilhvax in Association With Praziquantel for Prevention of Clinical Recurrences of Schistosoma Haematobium | Yes |
| Schistosomiasis | NCT00403611 | Evaluation of Praziquantel Dosage for Treatment of Schistosomiasis in Brazil | Yes |
| Schistosomiasis | NCT02178748 | Trial to Investigate the Effect of Schistosoma Mansoni Infection on the Response to Vaccination With MVA85A in BCG-vaccinated African Adolescents | Yes |
| Schistosomiasis | NCT01529710 | Safety and Efficacy of Mirazid for Schistosomiasis Treatment |  |
| Schistosomiasis | NCT01132248 | Activity of Mefloquine Against Urinary Schistosomiasis |  |
| Schistosomiasis | NCT03041766 | Study of Safety and Immune Response of the Sm14 Vaccine in Adults of Endemic Regions |  |
| Snake Bite Envenomation | NCT00639951 | Study to Evaluate the Efficacy of Two Treatment Schemes With Antivipmyn for the Treatment of Snake Bite Envenomation |  |
| Snake Bite Envenomation | RBR-3h33wy | Ciprofloxacin effectiveness of the assessment to prevent bacterial infection of patients victims of accidents with snake in the Brazilian Amazon |  |
| Snake Bite Envenomation | NCT02570347 | Routine Antibiotic vs. Directed Antibiotic Treatment in Snake Bite |  |
| Snake Bite Envenomation | NCT02877498 | A Study to Compare Adaptive Support Ventilation vs. Volume Controlled Ventilation for Management of Respiratory Failure in Patients With Neuroparalytic Snake Envenomation |  |
| Snake Bite Envenomation | SLCTR/2016/015 | A dose finding study in Hump-nosed pit viper bites with new antivenom |  |
| Snake Bite Envenomation | ACTRN12612001062819 | A Phase I/Phase II randomized controlled trial (RCT) of a new antivenom, compared to the currently used CSL taipan antivenom, for the treatment of the effects of Papuan taipan bite |  |
| Snake Bite Envenomation | RBR-9j7qqr | Dressing with product derived from snake venom to treat wounds in the legs |  |
| Snake Bite Envenomation | RBR-9mbdj3 | Safety assessment and dose of fibrin sealant derived from snake venom in the treatment of wounds. |  |
| Snake Bite Envenomation | NCT01284855 | Comparison of Two Dose Regimens of Snake Antivenom for the Treatment of Snake Bites Envenoming in Nepal |  |
| Snake Bite Envenomation | NCT00636116 | Phase 3 Multicenter Comparative Study to Confirm Safety and Effectiveness of the F(ab)2 Antivenom Anavip. |  |
| Snake Bite Envenomation | NCT01864200 | Randomized, Double-Blind, Placebo-Controlled Study: CroFab vs Placebo for Copperhead Snake Envenomation |  |
| Snake Bite Envenomation | NCT01337245 | Emergency Treatment of Coral Snake Envenomation With Antivenom |  |
| Soil Transmitted Helminthiases | NCT02397772 | Impact of Alternative Treatment Strategies and Delivery Systems for Soil-transmitted Helminths in Kenya |  |
| Soil Transmitted Helminthiases | NCT01308268 | Management of Soil-transmitted Helminthiasis and Strongyloidiasis |  |
| Soil Transmitted Helminthiases | NCT02102321 | Does Treating Hookworm Improve Productivity of Small Subsistence Farmers |  |
| Soil Transmitted Helminthiases | PER-028-13 | Post-partum Deworming and Infant Growth |  |
| Soil Transmitted Helminthiases | ISRCTN86603231 | Dose-finding of oxantel pamoate in school-aged children infected with Trichuris trichiura on Pemba, United Republic of Tanzania | Yes |
| Soil Transmitted Helminthiases | NCT01658774 | Impact of Repeated Anthelmintic Treatment on the Risk of Malaria in Kenyan School Children | Yes |
| Soil Transmitted Helminthiases | NCT01327469 | The Efficacy of Five Anthelmintic Regimes Against Trichuris Trichiura Infections in Schoolchildren in Jimma, Ethiopia | Yes |
| Soil Transmitted Helminthiases | NCT01087099 | A Multinational Trial of the Efficacy of Albendazole Against Soil-transmitted Nematode Infections in Children | Yes |
| Soil Transmitted Helminthiases | NCT01050452 | Safety and Efficacy of Drug Combinations Against Trichuris Trichiura | Yes |
| Soil Transmitted Helminthiases | NCT02034162 | A Study to Assess the Efficacy and Safety of Mebendazole for the Treatment of Helminth Infections in Pediatric Participants | Yes |
| Soil Transmitted Helminthiases | NCT01379326 | Monitoring the Efficacy of Anthelmintics for the Treatment of Soil Transmitted Helminths P2 | Yes |
| Soil Transmitted Helminthiases | NCT00367627 | Relative Efficacy of Two Regimens of Ante-helminthic Treatment | Yes |
| Soil Transmitted Helminthiases | NCT01192802 | Efficacy of Albendazole to Treat Intestinal Helminths and Its Effect on Gut Microflora | Yes |
| Soil Transmitted Helminthiases | NCT02509481 | Repeat Ivermectin Mass Drug Administrations for Control of Malaria: a Pilot Safety and Efficacy Study |  |
| Soil Transmitted Helminthiases | NCT01350271 | Comparative Efficacy of Different Mebendazole Polymorphs in the Treatment of Soil-transmitted Helminth Infections |  |
| Soil Transmitted Helminthiases | NCT02675140 | Effect of Hookworm Elimination and Vitamin A Intervention on Iron Status of Preschool Children in Sichuan, China |  |
| Soil Transmitted Helminthiases | NCT00765024 | Ivermectin Versus Albendazole for Chronic Strongyloidiasis |  |
| Soil Transmitted Helminthiases | NCT00469989 | Randomised Placebo Controlled Study of Effects of Therapeutic Hookworm Infection in Asthma |  |
| Soil Transmitted Helminthiases | NCT00671138 | Inoculating Celiac Disease Patients With the Human Hookworm Necator Americanus: Evaluating Immunity and Gluten-sensitivity |  |
| Soil Transmitted Helminthiases | NCT00659997 | Efficacy Albendazole and Levamisole Against STH on Unguja |  |
| Soil Transmitted Helminthiases | NCT01570504 | Multiple Versus Single Dose of Ivermectin for the Treatment of Strongyloidiasis |  |
| Taeniasis/Cysticercosis | NCT02945527 | Treatment of Peri-calcification Edema in Neurocysticercosis (NCC) |  |
| Taeniasis/Cysticercosis | NCT02947581 | Sub Arachnoid Neurocysticercosis Treatment Outcome (SANTO) |  |
| Taeniasis/Cysticercosis | NCT02243644 | Effects of 2 Different Duration of Albendazole Therapy in Patients With Neurocysticercosis in Brain â‰¤ 5 Lesions on CT | Yes |
| Taeniasis/Cysticercosis | NCT00441285 | Neurocysticercosis: Combined Treatment With Praziquantel (PZQ) and Albendazole (ABZ) |  |
| Taeniasis/Cysticercosis | NCT00290823 | Corticosteroids to Reduce Frequency of Seizures in Neurocysticercosis Patients |  |
| Taeniasis/Cysticercosis | PER-082-13 | Empirical and Targeted Treatment for Taenia Solium Solitaria (Peru) |  |
| Trachoma | NCT02754583 | Sanitation, Water, and Instruction in Face-washing for Trachoma |  |
| Trachoma | ACTRN12606000360516 | Impact of Annual Targeted Azithromycin Treatment on Infectious Trachoma and Susceptibility to Reinfection | Yes |
| Trachoma | ACTRN12615001199505 | A field trial of co-administration of azithromycin and ivermectin mass drug administration for scabies and trachoma. |  |
| Trachoma | NCT00792922 | Partnership for Rapid Elimination of Trachoma | Yes |
| Trachoma | NCT02048007 | Mortality Reduction After Oral Azithromycin: Morbidity Study |  |
| Trachoma | NCT00322972 | Trachoma Amelioration in Northern Amhara (TANA) |  |
| Trachoma | NCT01202331 | Tripartite International Research for the Elimination of Trachoma |  |
| Trachoma | NCT01586169 | Safety of the Co-administration of Three Drugs for Trachoma and Lymphatic Filariasis Elimination |  |
| Trachoma | NCT00522912 | A Trial of Epilation Verses Surgery for Minor Trichiasis |  |
| Trachoma | NCT00618449 | Impact of Two Alternative Dosing Strategies for Trachoma Control in Niger |  |
| Trachoma | NCT01949454 | Fluorometholone as Ancillary Therapy for TT Surgery |  |
| Trachoma | NCT01767506 | A Surveillance and Azithromycin Treatment for Newcomers and Travelers Evaluation: The ASANTE Trial |  |
| Trachoma | NCT01178762 | The Effect of Oral Azithromycin in the Treatment of Chlamydial Conjunctivitis |  |
| Yaws | PACTR201306000520103 | Single dose oral Azithromycin versus injection Benzathine benzylpenicillin in the treatment of yaws- A randomized noninferiority trial in some endemi |  |
| Yaws | NCT02344628 | Comparison of Two Different Doses of Azithromycin for Treatment of Yaws | Yes |
| Yaws | NCT01382004 | Single-dose Azithromycin for the Treatment of Yaws | Yes |
| Yaws | NCT01955252 | Effect of WHO-yaws Elimination Strategy in Lihir Island, Papua New Guinea |  |
| Yaws | NCT02775617 | Azithromycin - Ivermectin Mass Drug Administration for Skin Disease |  |
