# Supplementary material for: Neglected tropical diseases in children: An assessment of gaps in research prioritization
Source: PLoS Negl Trop Dis. 2019 Jan 29;13(1):e0007111. doi: 10.1371/journal.pntd.0007111 (PMC6368333; doi:10.1371/journal.pntd.0007111)
Supplement: S2 Table — (DOCX) [file pntd.0007111.s006.docx]

| **Condition** | **WHO-Recommended Therapies** | **Adequately Labeled for Use in Children** | **Age Range** | **Pediatric Formulation Available** |
| --- | --- | --- | --- | --- |
| Buruli ulcer | Rifampin and streptomycin | Yes | Rifampin: 1 months and older  Streptomycin: in infants | Rifampin: Yes  Streptomycin: N/A (IV medication) |
|  | Rifampin and clarithromycin | Yes | Rifampin: 1 months and older Clarithromycin: 6 months and older | Rifampin: Yes  Clarithromycin: Yes |
| Chagas (American trypanosomiasis) | Benznidazole | Yes | 2 to 12 years and older | Yes (can be made into a “slurry”) |
|  | Nifurtimox | No | N/A | N/A |
| Dengue | Supportive care | N/A | N/A | N/A |
| Chikungunya | Supportive care | N/A | N/A | N/A |
| Dracunculiasis (guinea-worm disease) | Supportive care | N/A | N/A | N/A |
| Echinococcosis |  |  |  |  |
| Cystic  echinococcosis | Surgery | N/A | N/A | N/A |
| Alveolar  echinococcosis | Surgery and albendazole | Albendazole: Yes | Albendazole: 6 years and older | No |
| Foodborne trematodiases |  |  |  |  |
| Fascioliasis | Triclabendazole | No | N/A | N/A |
| Opisthorchiasis | Praziquantel | Yes | 4 years and older | No |
| Clonorchiasis | Praziquantel | Yes | 4 years and older | No |
| Paragonimiasis | Triclabendazole | No |  |  |
|  | Praziquantel | Yes | 4 years and older | No |
| Human African Trypanosomiasis (sleeping sickness) |  |  |  |  |
| Stage one  (subcutaneous,  blood, and lymph  tissue) | Pentamidine | Yes | 16 years and older | No |
|  | Suramin | No |  |  |
| Stage two (central  nervous system) | Eflornithine and Nifurtimox | Eflornithine: Yes  Nifurtimox: No | Eflornithine: 12 years and older | N/A (cream) |
|  | Melarsoprol | No | N/A | N/A |
| Leishmaniasis |  |  |  |  |
| Visceral  leishmaniasis | Meglumine antimoniate | No | N/A | N/A |
|  | Sodium stibogluconate | No | N/A | N/A |
|  | Amphotericin B deoxycholate* | Yes | 2 years and older | N/A (IV medication) |
|  | Pentamidine isethionate* | Yes | 10 years and older | N/A (IV medication) |
| Cutaneous  leishmaniasis |  |  |  |  |
| Old World | Paromomycin ointment | No | N/A | N/A |
|  | Fluconazole | Yes | 6 months and older | Yes |
|  | Cryotherapy | N/A | N/A | N/A |
|  | Thermotherapy | N/A | N/A | N/A |
| New World | Paromomycin ointment | No | N/A | N/A |
|  | Topical methylbenzethonium | No | N/A | N/A |
|  | Topical ketoconazole | Yes | 12 years and older | No |
|  | Miltefosine | Yes | 12 years and older | No |
|  | Cryotherapy | N/A | N/A | N/A |
|  | Thermotherapy | N/A | N/A | N/A |
| Mucocutaneous | Pentavalent antimonials | No | N/A | N/A |
|  | Pentavalent antimonials plus oral pentoxifylline | No | N/A | N/A |
|  | Amphotericin B deoxycholate | Yes | 2 years and older | N/A (IV medication) |
|  | Liposomal amphotericin B | Yes | 2 years and older | N/A (IV medication) |
|  | Miltefosine | Yes | 12 years and older | No |
| Leprosy (Hansen's disease) |  |  |  |  |
| Single Lesion | Rifampin | Yes | 1 month and older | Yes |
|  | Minocycline | Yes | 12 years and older | No |
| Paucibacillary | Rifampin and dapsone | Rifampin-Yes  Dapsone: No FDA label | Rifampin: 1 month and older  Dapsone: N/A | Rifampin: Yes  Dapsone: N/A |
| Multibacillary | Dapsone, Rifampin and Clofazimine | Rifampin: Yes  Dapsone: No FDA label  Clofazimine: No | Rifampin: 1 month and older  Dapsone: N/A  Clofazimine: N/A | Rifampin: Yes  Dapsone: N/A  Clofazimine: N/A |
| Lymphatic filariasis | Albendazole and Diethylcarbamazine *or* Ivermectin | Albendazole: Yes  Diethylcarbamazine: No  Ivermectin: yes | Albendazole: 6 years and older  Diethylcarbamazine: N/A  Ivermectin: 15 kg and heavier | Albendazole: No  Diethylcarbamazine: N/A  Ivermectin: No |
| Mycetoma, chromoblastomycosis, and other deep mycoses | Itraconazole | No | N/A | N/A |
|  | Itraconazole and Flucytosine | No | N/A | N/A |
|  | Locally applied heat therapy | N/A | N/A | N/A |
|  | Cryosurgery | N/A | N/A | N/A |
| Onchocerciasis (river blindness) | Ivermectin | Yes | 15 kg and heavier | No |
| Rabies | Immediate vaccination and rabies immune globulin | Not specified | N/A | N/A |
| Scabies and other ectoparasites | Permethrin 5% cream | Yes | 2 months and older | N/A (cream) |
|  | Malathion 5% cream | Yes | 6 years and older | N/A (cream) |
|  | Benzyl benzoate emulsion 10–25% cream | No | N/A | N/A |
|  | Sulphur ointment 5–10% | No | N/A | N/A |
|  | Ivermectin | Yes | 15 kg and heavier | No |
| Schistosomiasis | Praziquantel | Yes | 4 years and older | No |
|  | Oxamniquine | No | N/A | N/A |
| Soil Transmitted Helminthiases |  |  |  |  |
| Ascariasis | Pyrantel | No | N/A | N/A |
|  | Mebendazole | Yes | 2 years and older | Yes-chewable tablets |
|  | Levamisole | No | N/A | N/A |
|  | Piperazine | No | N/A | N/A |
|  | Albendazole | Yes | 6 years and older | No |
|  | Flubendazole | No | N/A | N/A |
| Hookworm | Mebendazole | Yes | 2 years and older | Yes-chewable tablets |
|  | Albendazole | Yes | 6 years and older | No |
|  | Pyrantel | No | N/A | N/A |
|  | Levamisole | No | N/A | N/A |
|  | Flubendazole | No | N/A | N/A |
| Trichuriasis | Mebendazole | Yes | 2 years and older | Yes-chewable tablets |
|  | Albendazole | Yes | 6 years and older | No |
|  | Flubendazole | No | N/A | N/A |
| Strongyloidiasis | Albendazole | Yes | 6 years and older | No |
|  | Mebendazole | Yes | 2 years and older | Yes-chewable tablets |
|  | Ivermectin | Yes | 15 kg and heavier | No |
| Snakebite envenoming | Antivenom (varies by region) | Not specified | N/A | N/A |
| Taeniasis/Cysticercosis | Praziquantel | Yes | 4 years and older | No |
|  | Niclosamide | No | N/A | N/A |
| Neurocysticercosis | Praziquantel and corticosteroids and/or anti-epileptic drugs (combined) | Praziquantel: Yes  Corticosteroids: Yes | Praziquantel: 4 years and older  Corticosteroids: All | Praziquantel: No  Corticosteroids: Yes |
|  | Albendazole and corticosteroids | Albendazole : Yes  Corticosteroids: Yes | Albendazole: 6 years and older  Corticosteroids: All | Albendazole: No  Corticosteroids: Yes |
| Trachoma | Azithromycin | Yes | 6 months and older | Yes |
| Yaws | Azithromycin | Yes | 6 months and older | Yes |
|  | Benzathine penicillin | Yes | All | N/A (IV medication) |

*Second line therapy
